# Supplementary material for: Preparative-scale synthesis of nonacene
Source: Nat Commun. 2022 Jan 11;13:223. doi: 10.1038/s41467-021-27809-0 (PMC8752783; doi:10.1038/s41467-021-27809-0)
Supplement: Supplementary file 1 — Supplementary Information [file 41467_2021_27809_MOESM1_ESM.pdf]

SUPPLEMENTARY INFORMATION

Preparative-scale synthesis of nonacene

Andrej Jančařík *, Jan Holec, Yuuya Nagata, Michal Šámal, Andre Gourdon*

Centre d'Elaboration et d'Etude Structurale (CEMES)-CNRS, Toulouse, France

Table of contents *Page number*

Supplementary methods 3

The synthesis of nonacene 1 5

Supplementary Figure 2. 1H and 13C NMR spectra of 5a/b – 7a/b 12

Supplementary Figure 3 13C CPMAS spectra of transformations 7a/b → 1 20

Supplementary Figure 4. IR spectra of transformations 7a/b → 1 22

Supplementary Figure 5. IR spectra measured in KBr 25

Supplementary Figure 6. Thermal Gravimetric Analysis of 7a/b 27

Supplementary Figure 7. MALDI MS Analysis of 1 generated in different conditions 28

Supplementary Figure 8. Theoretical calculation of Diels Alder reaction 35

Supplementary Figure 9. X-ray analysis of 5b 36

Supplementary Table 1. Crystal data and structure refinement for 5b 37

Supplementary Table 2. Atomic coordinates (x 104) and equivalent isotropic displacement parameters (Å2x 103) for 5b 39

Supplementary Table 4. Anisotropic displacement parameters (Å2x 103) for 5b. 47

Supplementary references 50

Supplementary methods

General information

Starting compounds, catalysts and solvents were purchased from Sigma-Aldrich and TCI. Flash column chromatography was performed by using silica gel (60 Å pore size, 40-63 µm Merck). The reactions were monitored by thin layer chromatography (TLC) on silica gel-coated plates (Merck 60 F_{254}). The NMR spectroscopic data in solution were recorded with Bruker Avance 300 MHz and 500 MHz instruments and were calibrated by using the residual undeuterated solvent as an internal reference (CD_2Cl_2 at δH = 5.33 ppm, δC = 53.84 ppm; $CDCl_3$ at δH = 7.26 ppm, δC = 77.16 ppm; tetrachloroethane-d_2 at δH = 6.00 ppm, δC = 73.78 ppm). Chemical shifts are reported in parts per million (ppm) on the δ scale and coupling constants (J) are in Hertz (Hz). The abbreviations used to describe the multiplicities are s = singlet, dd = doublet of doublets ddd = doublet of doublet of doublet. Mass spectra were recorded at the Service Commun de Spectrometrie de Masse of University Paul Sabatier (Toulouse 3), Toulouse (France) and CP MAS NMR were recorded at the Laboratoire de Chimie de Coordination on a Bruker Avance IIIHD 400 spectrometer equipped with a 2.5 mm probe. Samples were spun at 14 kHz at the magic angle using ZrO_2 rotors. ^{13}C-CP/MAS were recorded with a recycle delay of 1.5 s and a contact time of 3 ms. All chemical shifts for ^{13}C are relative to TMS. Thermal analyses (TG/DSC) were carried out on a Setaram Labsys instrument under flowing helium (45 mL min^{-1}) with the heating rate of 10 °C min^{-1} from 30 °C to 550 °C. The samples (5 mg) were contained in 100 µL aluminum crucibles. IR spectra were measured on a PerkinElmer Spectrum 100 FT-IR spectrometer with samples as KBr pellets. UV - Vis spectra were recorded on a Varian Cary 5000 spectrophotometer.

Thermal Gravimetric Analysis

Ca 3-5 mg of samples were precisely weighed in a 100 µl alumina crucible and placed into a Setaram Labsys device. Before analysis, sample and analysis chamber were purged first in vacuo and then by flowing helium (45 ml/min) during 8 hours. The absence of oxygen was checked with a lambda analyser (Setnag JC15V) coupled with the Labsys device. Dual thermogravimetry (TG) and differential thermal analysis (DTA) of samples were made simultaneously using a ramp rate of 10 °C/min, from 30 °C to 550 °C and with a helium flow of 45 ml/min.

MS MALDI measurements:

Saturated solution of the carbonyl precursor **7a** or **7b** in dichloromethane was drop-casted on an indium-tin oxide (ITO) glass slide without any matrix to avoid possible side reactions of the later generated reactive nonacene molecules. After evaporation of the solvent, the coated ITO glass slide was heated in a glovebox at 200°C for 1 min. and then subjected to the MALDI chamber for the measurement. In the second experiment, the soluble precursor **7a** was dissolved in chlorobenzene and the solution was thoroughly degassed. This solution was heated at 200°C for 10 min. during a dark violet precipitate was formed. This heterogenous solution was drop-casted on an indium-tin oxide (ITO) glass slide without any matrix and dried. After evaporation of the solvent, the coated ITO glass slide was subjected to the MALDI chamber for the measurement. In a parallel experiment was the heterogenous solution mixed with 2,5-dihydroxy benzoic acid as a matrix and then drop-casted on an indium-tin oxide (ITO) glass slide and dried. After evaporation of the solvent, the coated ITO glass slide was subjected to the MALDI chamber for the measurement. ITO glass slides and cover slips were obtained from Bruker (Bremen, Germany). The laser spot was set at the small focus with a laser intensity 41% and calibrated on red phosphorus clusters.

FTIR spectra in KBr pellet

KBr pellets were prepared from 1 mg of **7a**, **7b**, respectively and 100 mg of anhydrous KBr. Compounds **7a**/**b** were converted to nonacene **1** by heating the pellets on a heating plate at 200 or 350°C°C for 30 s under inert atmosphere and then immediately measured under ambient conditions. Alternatively, 1 mg of **7a**/**7b** were heated at 200°C/350°C to give **1** and the pellets were prepared from **1.** Another solution was to use **1** obtained by precipitation from chlorobenzene as for Maldi MS (see above). In this case, it was not possible to remove traces of chlorobenzene from dried **1**. Oxidation products of acenes: pentacene and heptacene generated in solid state from their carbonyl precursors were dispersed in dichloromethane and stirred open to air and direct light for several hours. Then the solvent was evaporated and the solid residue was measured in KBr pellet.

Theoretical calculation:

Diels-Alder selectivity: To gain insight into the selectivity of the Diels-Alder reaction between diene and aryne, we conducted density functional theory (DFT) calculations of transition states

(TS) to form **5a**/**5b** using GRRM 17[i] with Gaussian 16 Rev. C.01.[1] The geometries of the TS were optimized with QST3 method at the B3LYP/6-31+G(d,p) level of theory, and the energies of TS were calculated by single point calculations at the M06-2X/6-31+G(d,p) level of theory with according to a previous paper dealing with the Diels-Alder reaction.[2] The polarizable continuum model (PCM) was used for the geometry optimization and the single point calculations to take into consideration the effect of the reaction solvent (acetonitrile). As shown in SI (page S28), 8 structures were determined as TS. Based on the calculated energies of TS, syn/anti ratio of **1** was estimated to 1.64/1, which is consistent with the observed stereoselectivity in the experiment (syn/anti = 2/1).

The synthesis of nonacene 1

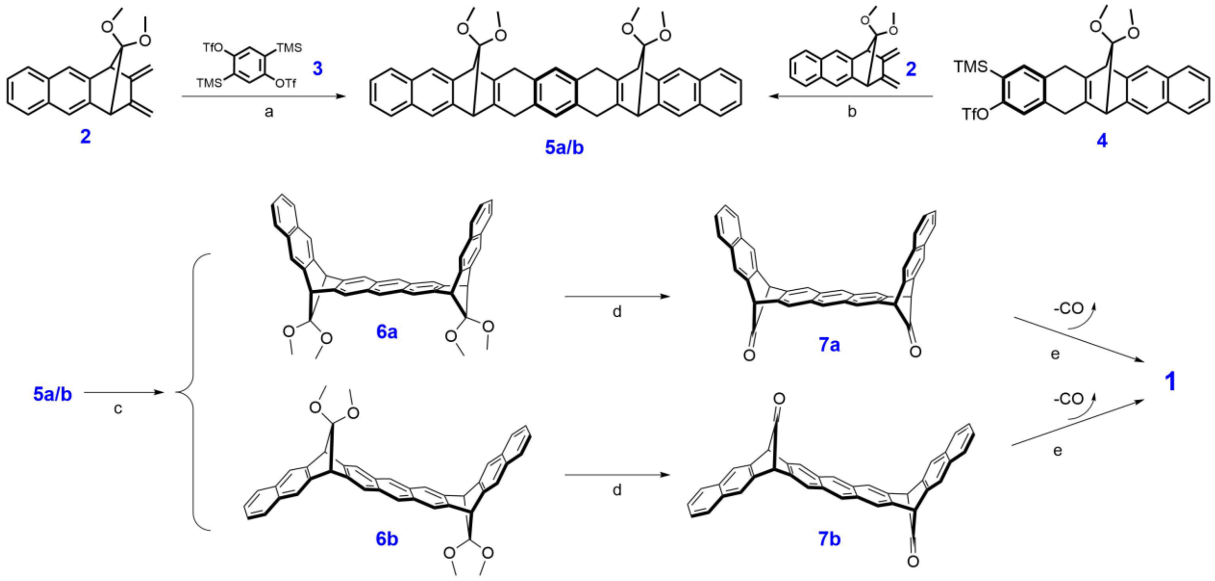

Supplementary Figure 1. Synthesis of nonacene 1

(1*R*, 4*S*)-11,11-dimethoxy-2,3-dimethylene-1,2,3,4-tetrahydro-1,4-methanoanthracene (2)

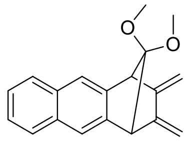

The diene **2** was prepared according to our previously published method and the ^{1}H NMR and ^{13}C NMR spectra were in agreement with our previous data.[3]

^{1}H NMR (300 MHz, CD_2Cl_2): 3.11 (3H, s), 3.33 (3H, s), 4.09 (2H, s), 5.12 (2H, s), 5.29 (2H, s), 7.39 – 7.49 (2H, m), 7.63 (2H, s), 7.74 – 7.80 (2H, m).

(13S)-15,15-dimethoxy-3-(trimethylsilyl)-5,6,13,14-tetrahydro-6,13-methanopentacen-2-yl trifluoromethanesulfonate (4)

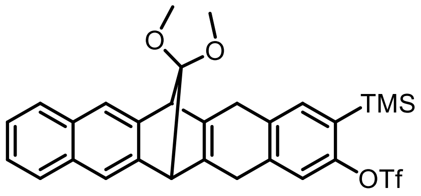

The aryne precursor **4** was prepared according to our previously published method and the ^{1}H NMR and ^{13}C NMR spectra were in agreement with our previous data.[4]

^{1}H NMR (300 MHz, CD_2Cl_2): 0.30 (9H, s), 3.08 (3H, s), 3.28 (3H, s), 3.32 – 3.45 (2H, m), 3.65 – 3.77 (2H, m), 4.00 (2H, s), 7.06 (1H, s), 7.26 (1H, s), 7.35 – 7.41 (2H, m), 7.59 (2H, s), 7.68 – 7.74 (2H, m).

(6S, 10R, 17S, 21R)-23,23,24,24-tetramethoxy-6,7,9,10,17,18,20,21-octahydro-6,21:10,17-dimethanononacene (5a) and (6S, 10S, 17R, 21R)-23,23,24,24-tetramethoxy-6,7,9,10,17,18,20,21-octahydro-6,21:10,17-dimethanononacene (5b)

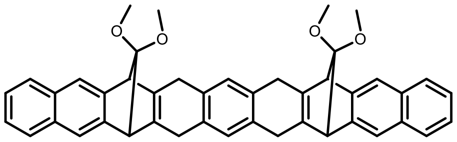

A well dried flask was charged with compound **4** (200 mg, 0.348 mmol, 1.0 equiv.), diene **2** (97 mg, 0.348 mmol, 1.0 equiv.) and CsF (106 mg, 0.699 mmol, 2 equiv.) under argon and then anhydrous CH_3CN (16 mL) and THF (3.2 mL) were added. The reaction mixture was stirred at room temperature overnight. After the evaporation of the solvent, the residue was purified by column chromatography on silica gel (gradient from chloroform to chloroform : aceton 20:1) was resolved first isomer **5b** (75 mg) from second isomer **5a** (140 mg) both as a white amorphous solid (combined yield 215 mg, 98 %). Both compounds were used directly in the next step without full characterization.

Isomer 5a:

^{1}H NMR (300 MHz, CD_2Cl_2): 3.06 (6H, s), 3.24 (6H, s), 3.26 – 3.32 (4H, m), 3.52 – 3.61 (4H, m), 3.95 (4H, s), 6.84 (2H, s), 7.35 – 7.40 (4H, m), 7.58 (4H, s), 7.68 – 7.73 (4H, m).
^{13}C NMR (126 MHz, CD_2Cl_2): not measured

Isomer 5b:

^{1}H NMR (300 MHz, CD_2Cl_2):): 3.07 (6H, s), 3.19 – 3.27 (4H, m), 3.29 (6H, s), 3.55 – 3.64 (4H, m), 3.96 (4H, s), 6.84 (2H, s), 7.31 – 7.36 (4H, m), 7.55 (4H, s), 7.64 – 7.69 (4H, m).
^{13}C NMR (126 MHz, CD_2Cl_2): not measured

(6S, 10R, 17S, 21R)-23,23,24,24-tetramethoxy-6,10,17,21-tetrahydro-6,21:10,17-dimethanononacene and (6a) and (6S, 10S, 17R, 21R)-23,23,24,24-tetramethoxy-6,10,17,21-tetrahydro-6,21:10,17-dimethanononacene (6b)

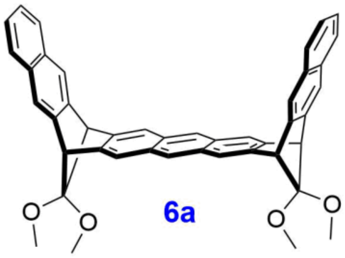

6a

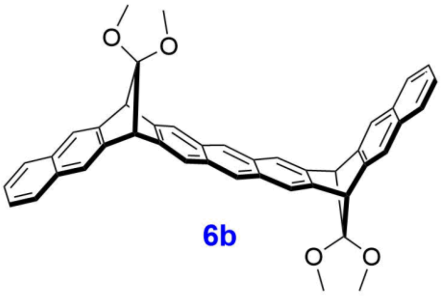

6b

Direct synthesis from 2 without isolation of 5a/b: A well-dried Schlenk flask was charged with diene **2** (200 mg, 0.72 mmol) and CsF (480 mg, 3.16 mmol, 4.0 equiv.) under argon and then anhydrous acetonitrile (16 mL) was added. The heterogenous mixture was cooled to 0°C and then solution of aryne precursor bis(trimethylsilyl)-1,4-phenylene bis(trifluoromethanesulfonate) (298 mg, 0.58 mmol, 0.8 equiv.) in anhydrous THF (4 mL) was added dropwise. The reaction was allowed to warm to room temperature overnight. Progress of the reaction was controlled by TLC, eluent (hexane - EtOAc 3:2). Reaction time depending on a scale of the reaction (1-3 days). After the evaporation of the solvent, the residue was chromatographed on silica gel (hexane : acetone 3:1) to get the desired product as a mixture of two regioisomers as a colorless solid. This mixture (146 mg, 0.23 mmol) was dissolved in anhydrous toluene (15 mL) under argon. The solution was cooled to 0°C and then DDQ (53 mg, 0.231 mmol, 2 equiv.) was added in one portion. The reaction mixture was stirred at 0°C for 10 min. and then 6 hours at room temperature. The volume of the reaction mixture was reduced to a half and the mixture was filtered over a frit S4. The solid was washed with toluene and finally with methanol to get the first isomer **6b** (40 mg) as a white solid. The mother liquor was evaporated and the residue was purified by chromatography on silica gel (hexane : acetone 3:1) to get the second isomer **6a** (82 mg) as a white solid. The ratio of isomers is 1:2 and combined yield is 54 % after two synthetic steps.

Synthesis by oxidation of 5a/b: The compound **5a** or **5b** (140 mg, 0.22 mmol) was dissolved in anhydrous toluene (17 mL) under argon. The solution was cooled to 0°C and then DDQ (111 mg, 0.488 mmol, 2.2 equiv.) was added in one portion. The reaction mixture was stirred at 0°C for 10 min. and then 6 hours at room temperature. The isomer **6b** was isolated by filtration the heterogenous reaction mixture over a frit S4. The collected solid was washed with toluene and then with methanol and finally dried to get the product **6b** as a white solid (130 mg, 94%).

Evaporation of the reaction mixture of the isomer **6a** followed by chromatography on silica gel (hexane : acetone 3:1) afforded the product as a white solid (129 mg, 94%).

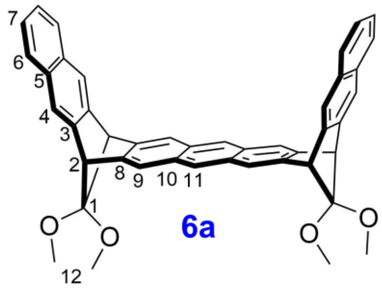

6a

^{1}H NMR (500 MHz, CD_2Cl_2): 3.23 (6H, s, *H12*), 3.26 (6H, s, *H12*), 4.71 (4H, s, *H2*), 7.33 - 7.36 (4H, m, *H7*), 7.68 – 7.71 (4H, m, *H6*), 7.70 (4H, s, *H4*), 7.77 (4H, s, *H9*), 8.12 (2H, s, *H11*).

^{13}C NMR (126 MHz, CD_2Cl_2): 51.54 (*C12*), 51.58 (*C12*), 55.20 (*C2*), 120.43 (*C9*), 120.87 (*C4*), 124.77 (*C1*), 125.96 (*C7*), 126.04 (*C11*), 128.14 (*C6*), 131.39 (*C10*), 133.08 (*C5*), 142.94 (*C8*), 143.61 (*C3*).

DCI MS: 627 ($[M + H]^+$).

HR DCI MS: calcd for $C_{44}H_{35}O_4$ 627.2530; found 627.2511.

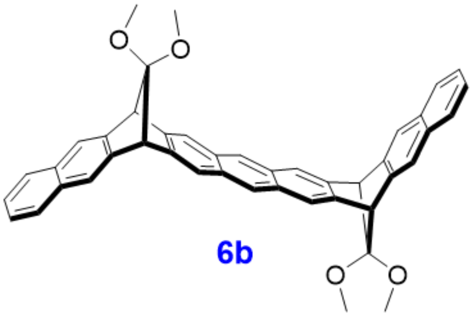

6b

^{1}H NMR (300 MHz, CD_2Cl_2): 3.21 (6H, s), 3.22 (6H, s), 4.71 (4H, s), 7.37 – 7.40 (4H, m), 7.72 – 7.77 (4H, m), 7.73 (4H, s), 7.77 (4H, s), 8.12 (2H, s).

^{13}C NMR (126 MHz, CD_2Cl_2): not measured due to the low solubility

DCI MS: 627 ($[M + H]^+$).

HR DCI MS: calcd for $C_{44}H_{35}O_4$ 627.2530; found 627.2523.

(6S, 10R, 17S, 21R)-6,10,17,21-tetrahydro-6,21:10,17-dimethanononacene-23,24-dione (7a)

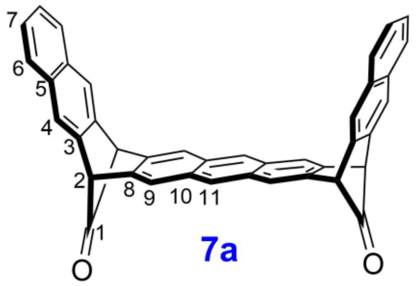

In a well dried Schlenk flask, **6a** (160 mg, 0.255 mmol) was dissolved in anhydrous dichloromethane (10 mL) under argon. Then trimethylsilyl iodide (109 µL, 0.766 mmol, 3 equiv.) was added dropwise and the homogeneous reaction mixture was stirred overnight at room temperature. Next day the heterogeneous reaction mixture was stirred on air at room temperature for 6 hours to complete the hydrolysis of formed iodo-methoxy intermediate. The product was collected by filtration over a glass frit filter S4, washed with mixture of solvents (hexane : dichloromethane, 4:1) to afford first portion of pure compound **7a** as a white solid. The mother liquor was evaporated and the residue was purified by chromatography on silica gel (hexane : acetone 3:1) to get the second portion of the product as a white solid. The combined yield was (130 mg, 95%).

^{1}H NMR (500 MHz, CD_2Cl_2): 4.99 (4H, s, *H2*), 7.41 - 7.44 (4H, m, *H7*), 7.78 – 7.81 (4H, m, *H6*), 7.93 (4H, s, *H4*), 8.04 (4H, s, *H9*), 8.36 (2H, s, *H11*).

^{13}C NMR (126 MHz, CD_2Cl_2): 57.55 (*C2*), 120.88 (*C9*), 121.22 (*C4*), 126.57 (*C7*), 126.76 (*C11*), 128.34 (*C6*), 131.61 (*C10*), 133.31 (*C5*), 137.61 (*C8*), 137.93 (*C3*), 194.46 (C=O).
CP MAS: 56.99 (bridgehead), 120.20-137.12 (aromatic), 193.26 and 197.73 (C=O).

IR (KBr pellet): 3056 m, 3012 m, 2954 m, 2922 m, 2853 m, 1793 s (C=O), 1610 w, 1582 w, 1499 m, 1441 w, 1420 m, 1401 w, 1339 w, 1283 w, 1265 w, 1207 w, 1175 wm, 1152 wm, 1135 m, 1094 w, 1019 w, 979 wm, 949 w, 916 ms, 880 ms, 820 w, 799 w, 782 w, 748 ms, 686 m, 590 w, 558 w, 524 wm, 492 wm, 474 m cm^{-1}.

DCI MS: 478 ([M – 2 x CO]$^+$).

HR DCI MS: calcd for $C_{38}H_{22}^+$ 478.1716 (M – 2xCO); found 478.1727.

(6S, 10S, 17R, 21R)-6,10,17,21-tetrahydro-6,21:10,17-dimethanononacene-23,24-dione (7b)

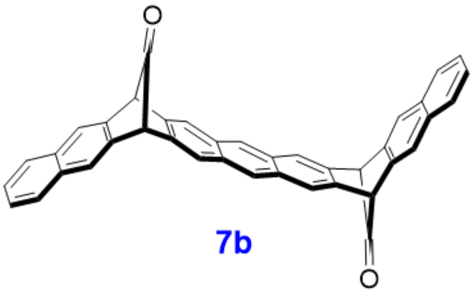

7b

In a well dried Schlenk flask, **6b** (25 mg, 39.89 µmol) was suspended in anhydrous dichloromethane (4 mL) under argon. Then trimethylsilyl iodide (23 µL, 159.6 µmol, 4 equiv.) was added dropwise and the heterogenous reaction mixture was stirred overnight at room temperature. Next day the heterogeneous reaction mixture was stirred on air at room temperature for 6 hours to complete the hydrolysis of formed iodo-methoxy intermediate. The product was collected by filtration over a glass frit filter S4, washed with mixture of solvents (dichloromethane : acetone, 4:1) to afford pure compound **7b** (20 mg, 94 %) as a white solid.

CP MAS: 55.83 (bridgehead), 119.23-136.01 (aromatic), 195.55 (C=O).

IR (KBr pellet) cm^{-1}: 3052 wm, 3012 wm, 1781 s (C=O), 1649 w, 1609 w, 1581 w, 1500 wm, 1440 w, 1420 m, 1397 wm, 1341 w, 1284 w, 1265 w, 1213 w, 1208 w, 1184 wm, 1144 wm, 1136 m, 1020 w, 979 wm, 938 w, 929 m, 911 m, 891 m, 881 m, 818 w, 800 w, 753 m, 709 w, 666 wm, 656 w, 607 w, 558 w, 529 w, 477 m.

DCI MS: 478 ([M – 2 x CO]$^+$).

HR DCI MS: calcd for $C_{38}H_{22}^+$ 478.1716 (M – 2xCO); found 478.1731.

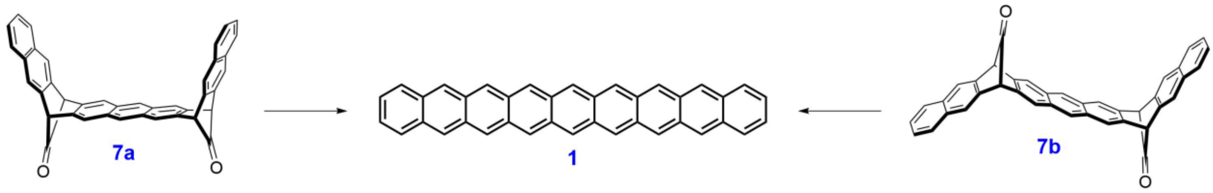

Nonacene (1)

Nonacene was obtained by heating **7a** or **7b** in the solid state at 200°C or 350°C under vacuum for 15 min.

IR (KBr pellet) cm^{-1}: 3045 m, 3018 m, 2925 w, 1924 wb, 1790 wb, 1624 w, 1507 w, 1500 w, 1450 w, 1445 w, 1418 w, 1362 w, 1296 w, 1269 w, 1162 w, 1123 w, 1104 w, 997 w, 952 w, 904 s, 735 ms, 621 w, 538 w, 466 m.

CP MAS: 125.5 - 128.8

MALDI MS: calcd for $C_{38}H_{22}$ 478.1722; found 478.179 - 478.335 (for details see page S28-S31).

HR MALDI MS: calcd for $C_{38}H_{22}$ 478.1722; found 478,1735 (measured without matrix) and 478,1718 (measured with DHB as a matrix).

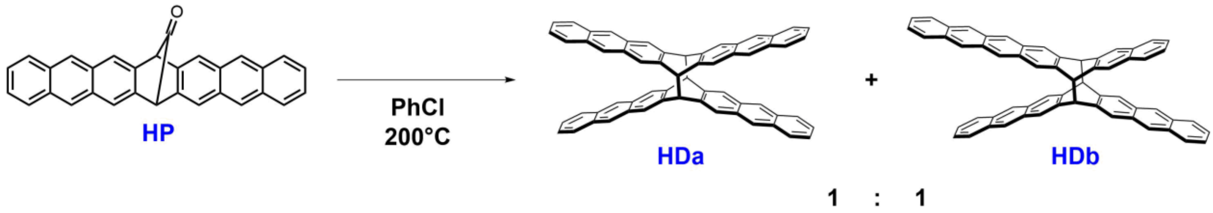

A well dried Young pressure tube was charged with heptacene carbonyl precursor[3] (5 mg) and anhydrous chlorobenzene (5 mL). The milky heterogenous mixture was three times thoroughly degassed and then heated to 200°C for 10 min. When the temperature reached 200°C, the reaction mixture become homogenous, and the colour turned to vivid yellow. The chlorobenzene was evaporated *in vacuo* to afford an orange solid which was washed several times with acetonitrile. The ^{1}H NMR confirmed formation of two heptacene dimers **HDa** and **HDb** in a ratio 1:1.

^{1}H NMR of HDa (300 MHz, tetrachloroethane-d_2): 5.33 (4H, s), 7.26-7.30 (8H, m), 7.72 (8H, s), 7.77-7.80 (8H, m), 8.11 (8H, s).

Supplementary Figure 2. 1H and 13C NMR spectra of 5a/b – 7a/b

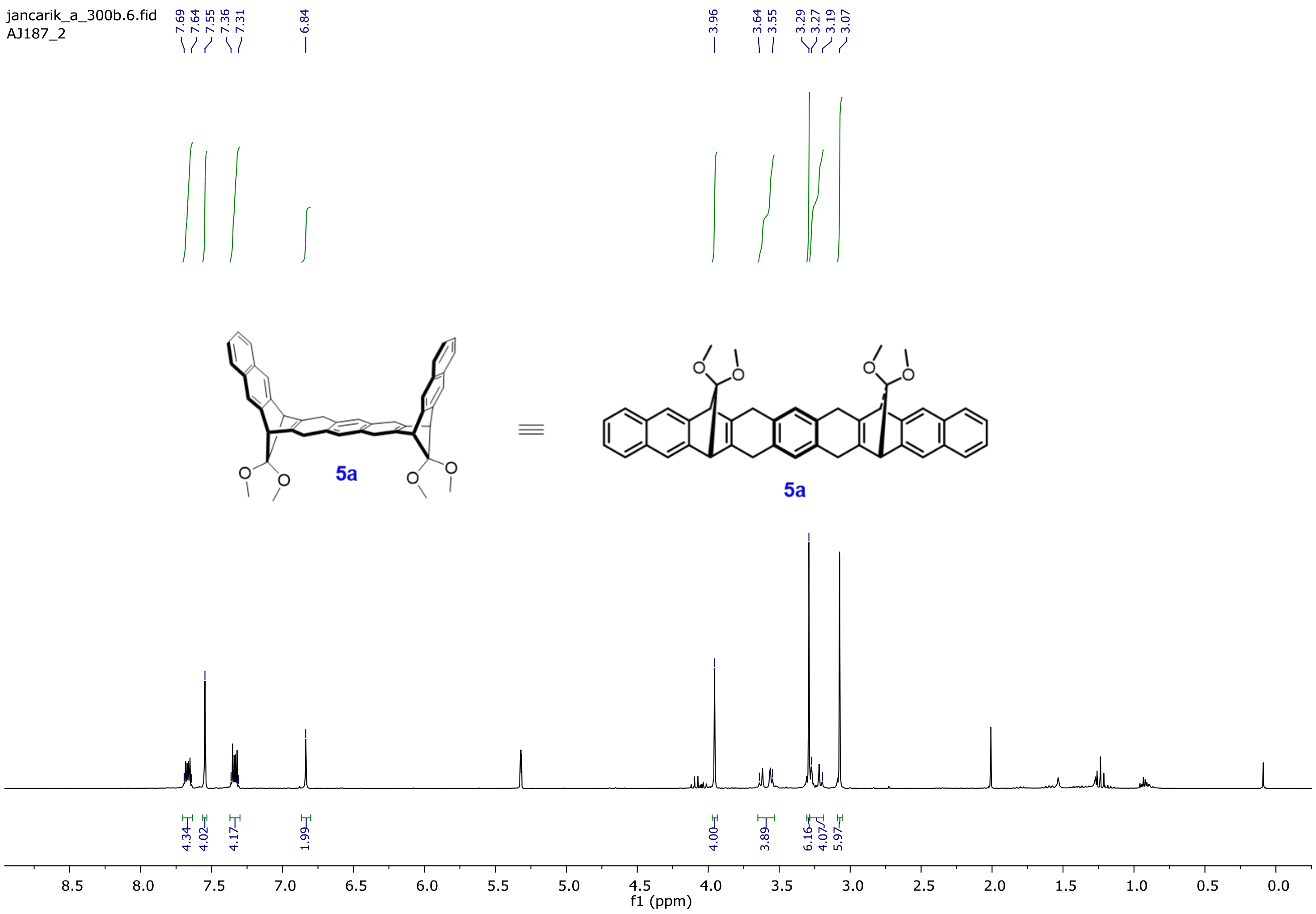

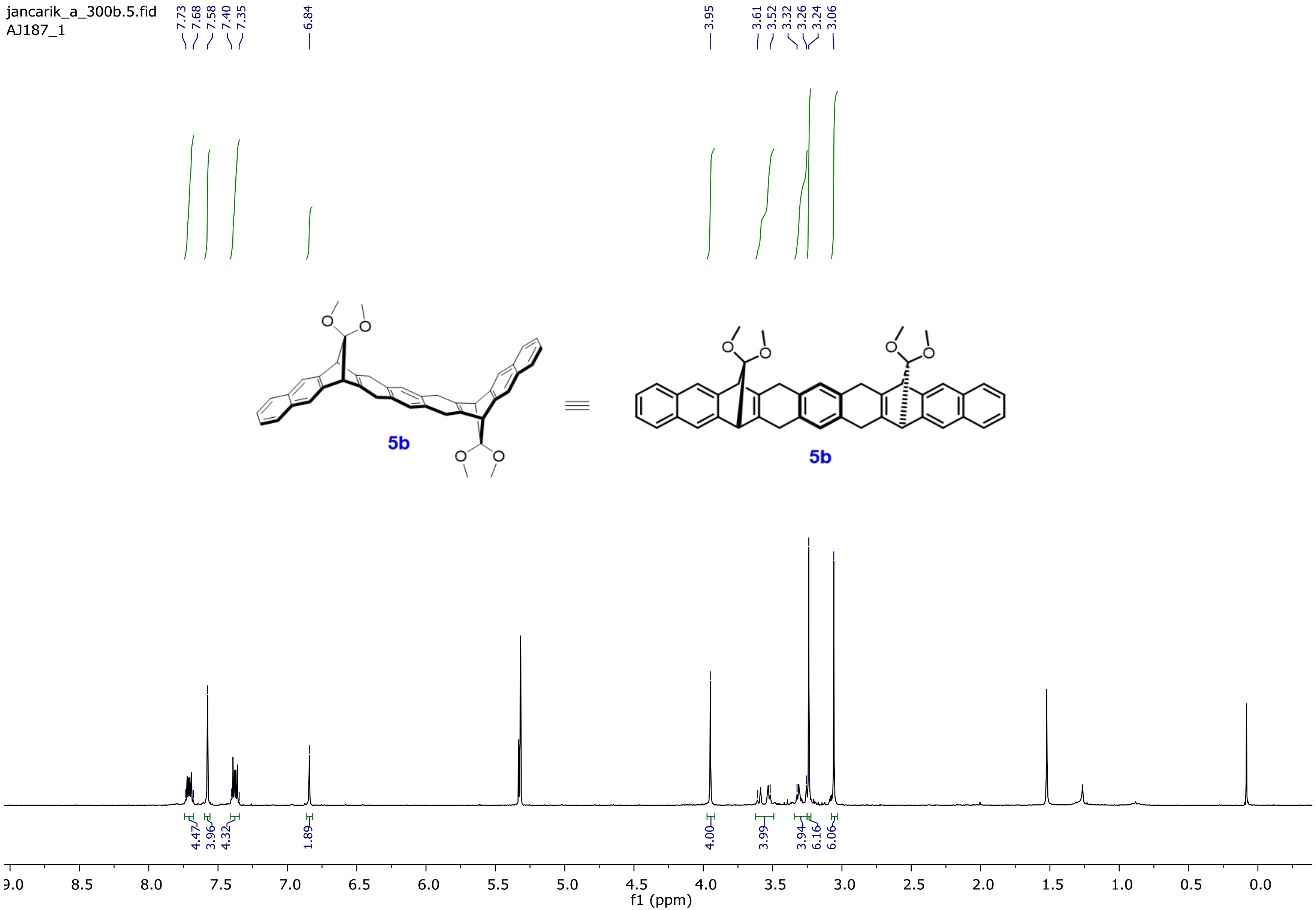
jancarik_a_300b.5.fid
AJ187_1
7.73
7.68
7.58
7.40
7.35
6.84
3.95
3.61
3.52
3.32
3.26
3.24
3.06
5b
5b
4.47
3.96
4.32
1.89
4.00
3.99
3.94
6.16
6.06
9.0
8.5
8.0
7.5
7.0
6.5
6.0
5.5
5.0
4.5
4.0
3.5
3.0
2.5
2.0
1.5
1.0
0.5
0.0
f1 (ppm)

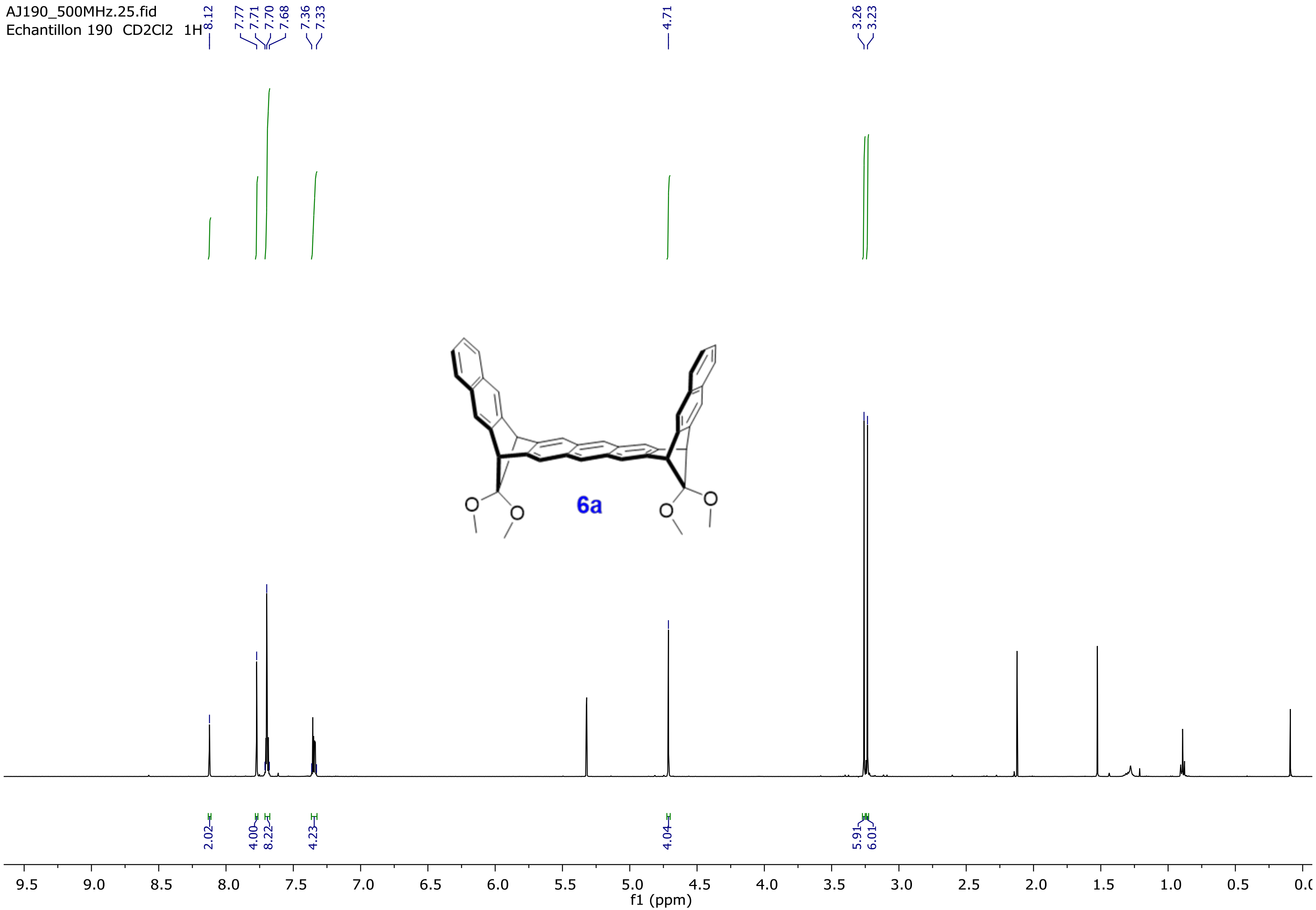
AJ190_500MHz.25.fid
Echantillon 190 CD2Cl2 1H
8.12
7.77
7.71
7.70
7.68
7.36
7.33
4.71
3.26
3.23
6a
2.02
4.00
8.22
4.23
4.04
5.91
6.01
9.5 9.0 8.5 8.0 7.5 7.0 6.5 6.0 5.5 5.0 4.5 4.0 3.5 3.0 2.5 2.0 1.5 1.0 0.5
f1 (ppm)

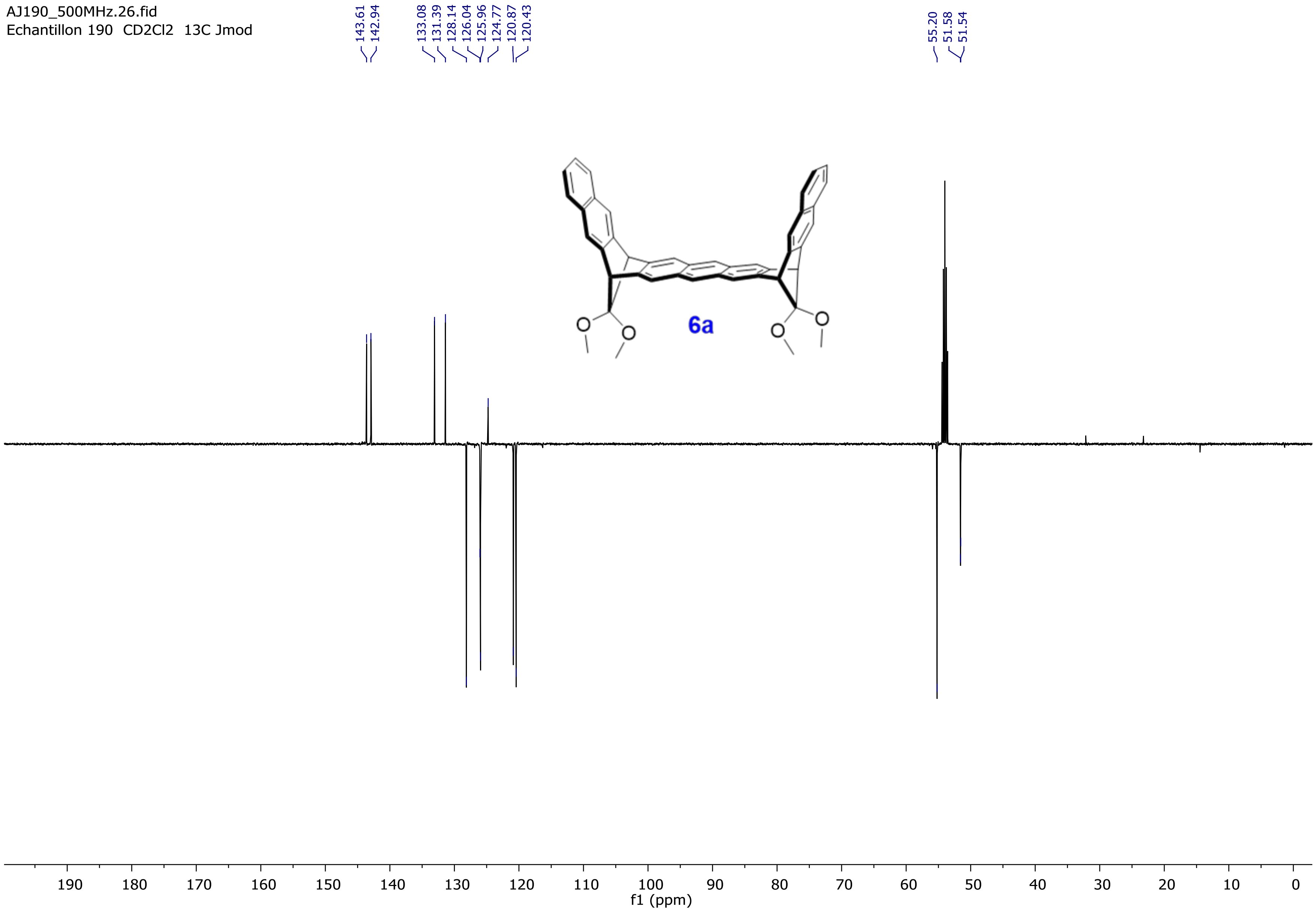
AJ190_500MHz.26.fid
Echantillon 190 CD2Cl2 13C Jmod
143.61
142.94
133.08
131.39
128.14
126.04
125.96
124.77
120.87
120.43
55.20
51.58
51.54
6a
190 180 170 160 150 140 130 120 110 100 90 80 70 60 50 40 30 20 10 0
f1 (ppm)

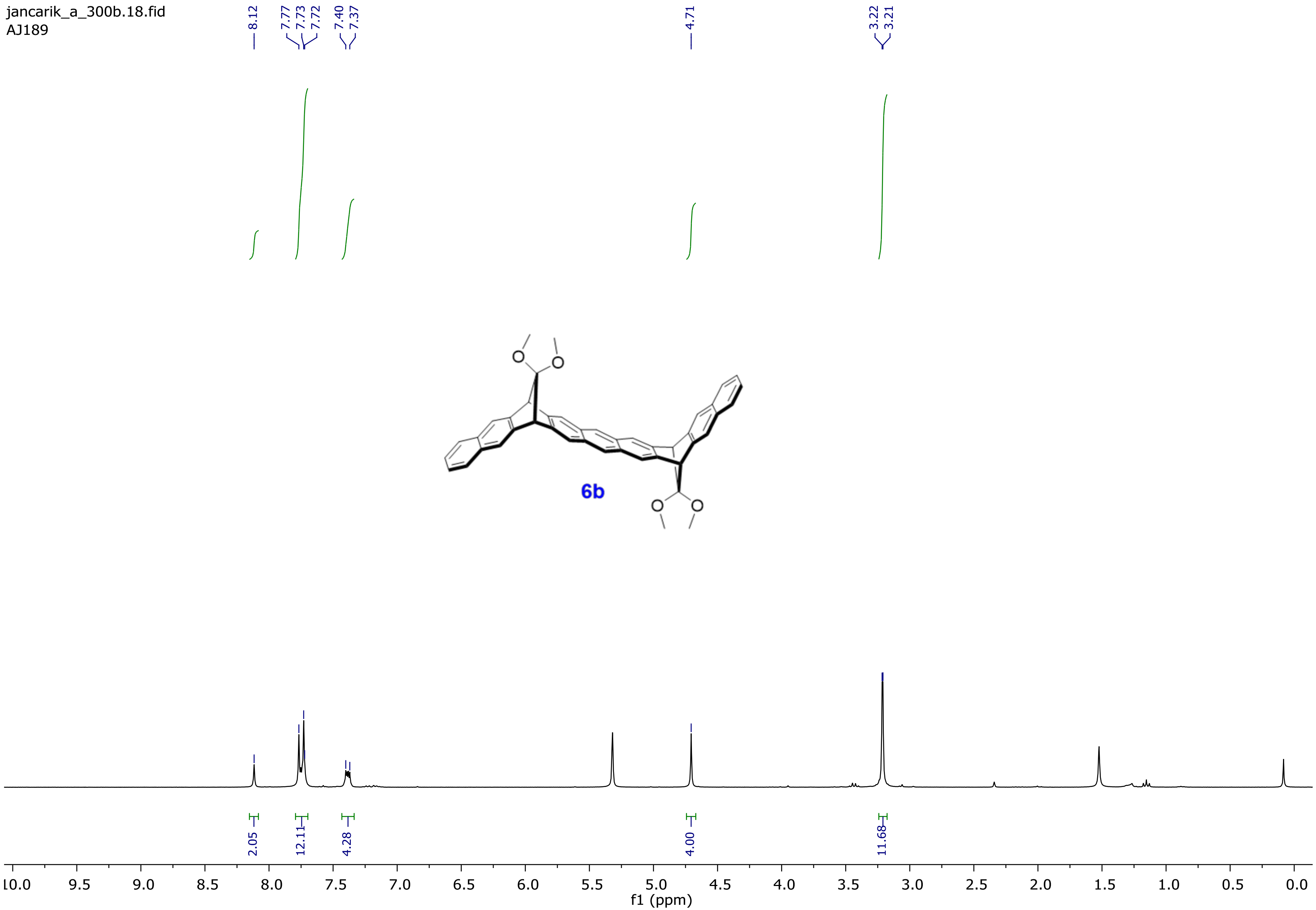
jancarik_a_300b.18.fid
AJ189
8.12
7.77
7.73
7.72
7.40
7.37
4.71
3.22
3.21
6b
2.05
12.11
4.28
4.00
11.68
10.0 9.5 9.0 8.5 8.0 7.5 7.0 6.5 6.0 5.5 5.0 4.5 4.0 3.5 3.0 2.5 2.0 1.5 1.0 0.5 0.0
f1 (ppm)

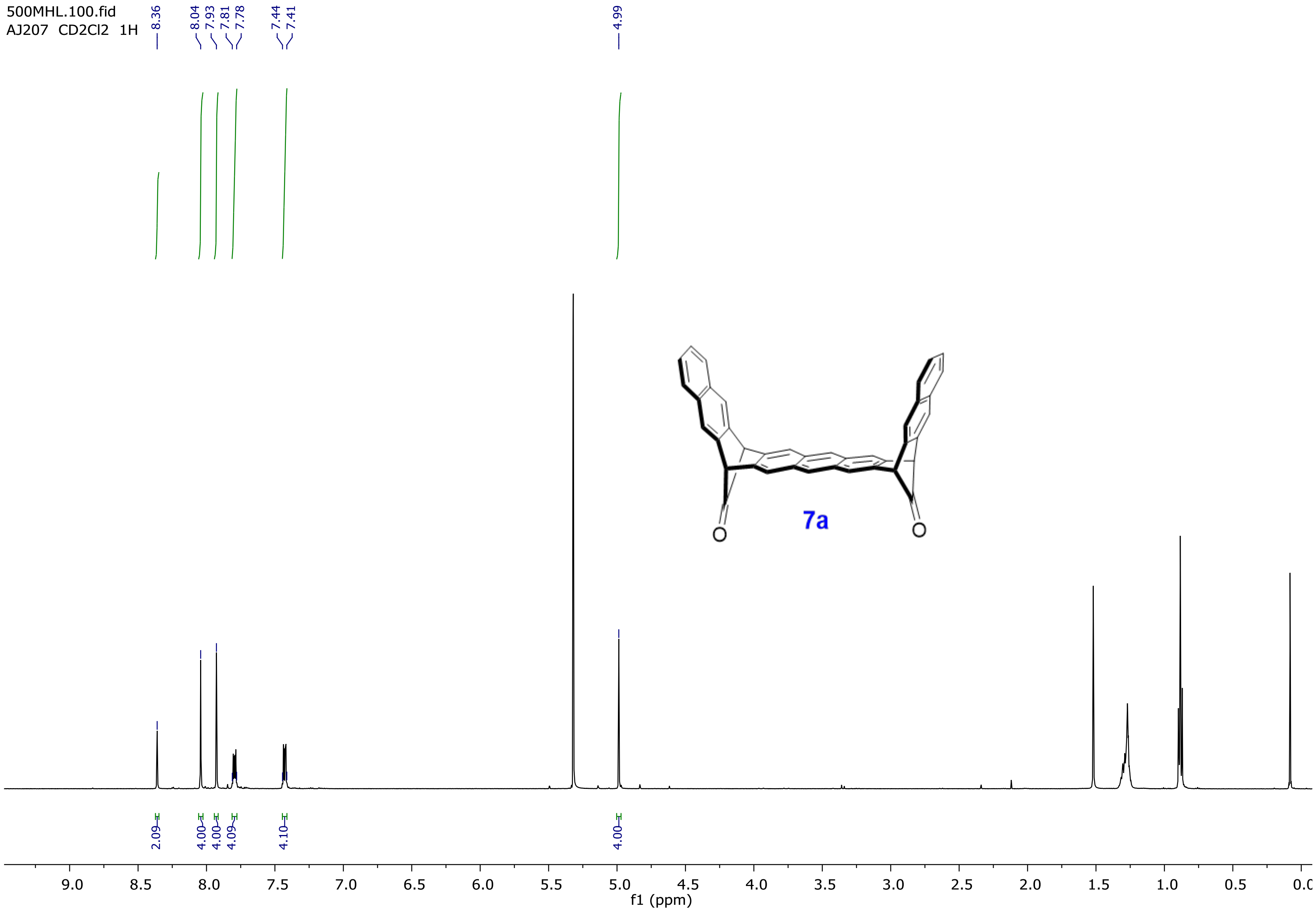
500MHL.100.fid
AJ207 CD2Cl2 1H
8.36
8.04
7.93
7.81
7.78
7.44
7.41
4.99
7a
O
O
2.09
4.00
4.00
4.09
4.10
4.00
9.0
8.5
8.0
7.5
7.0
6.5
6.0
5.5
5.0
4.5
4.0
3.5
3.0
2.5
2.0
1.5
1.0
0.5
f1 (ppm)

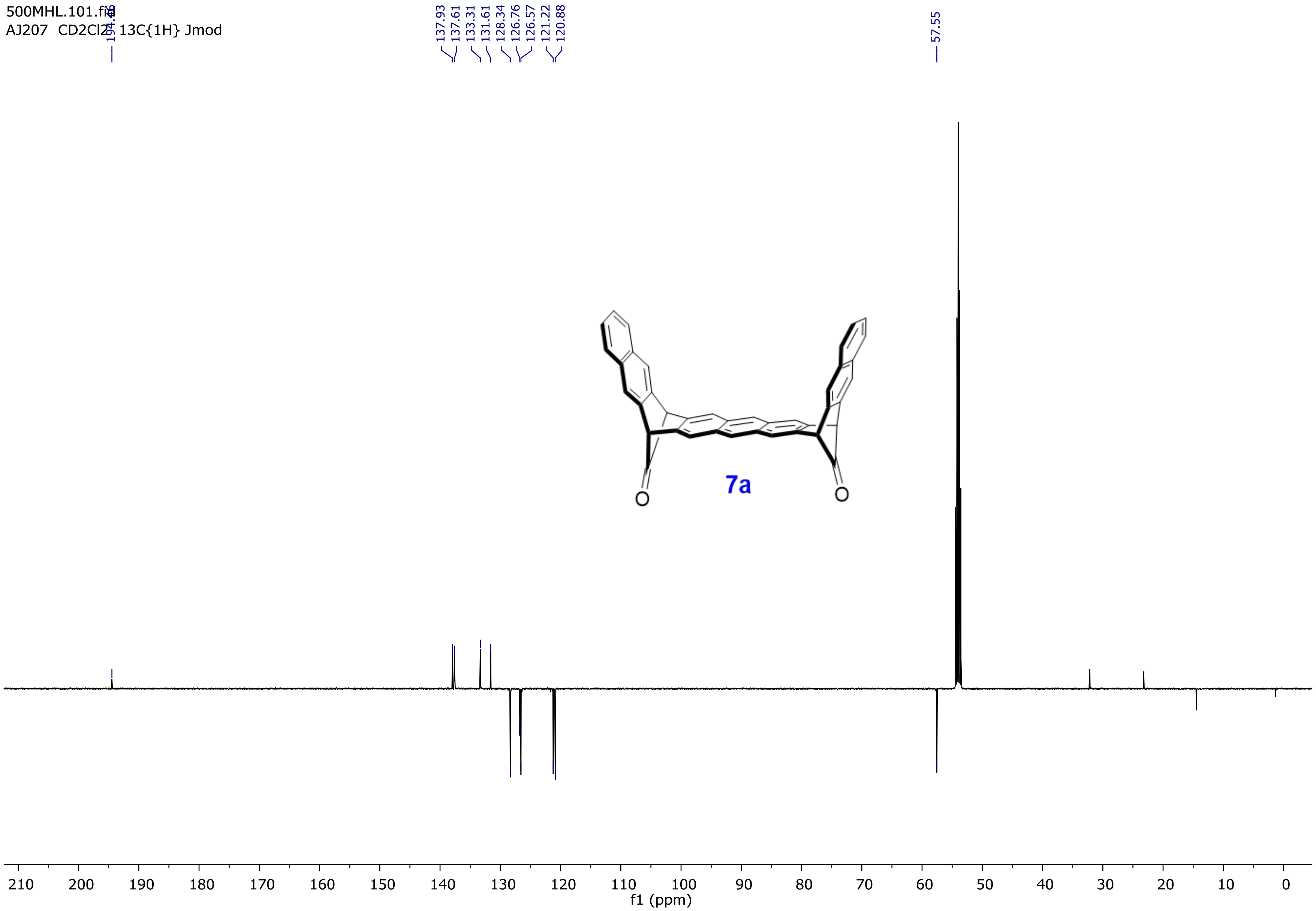

500MHL.101.fid
AJ207 CD2Cl2 13C{1H} Jmod
194.46
137.93
137.61
133.31
131.61
128.34
126.76
126.57
121.22
120.88
57.55
7a
210
200
190
180
170
160
150
140
130
120
110
100
90
80
70
60
50
40
30
20
10
0
f1 (ppm)

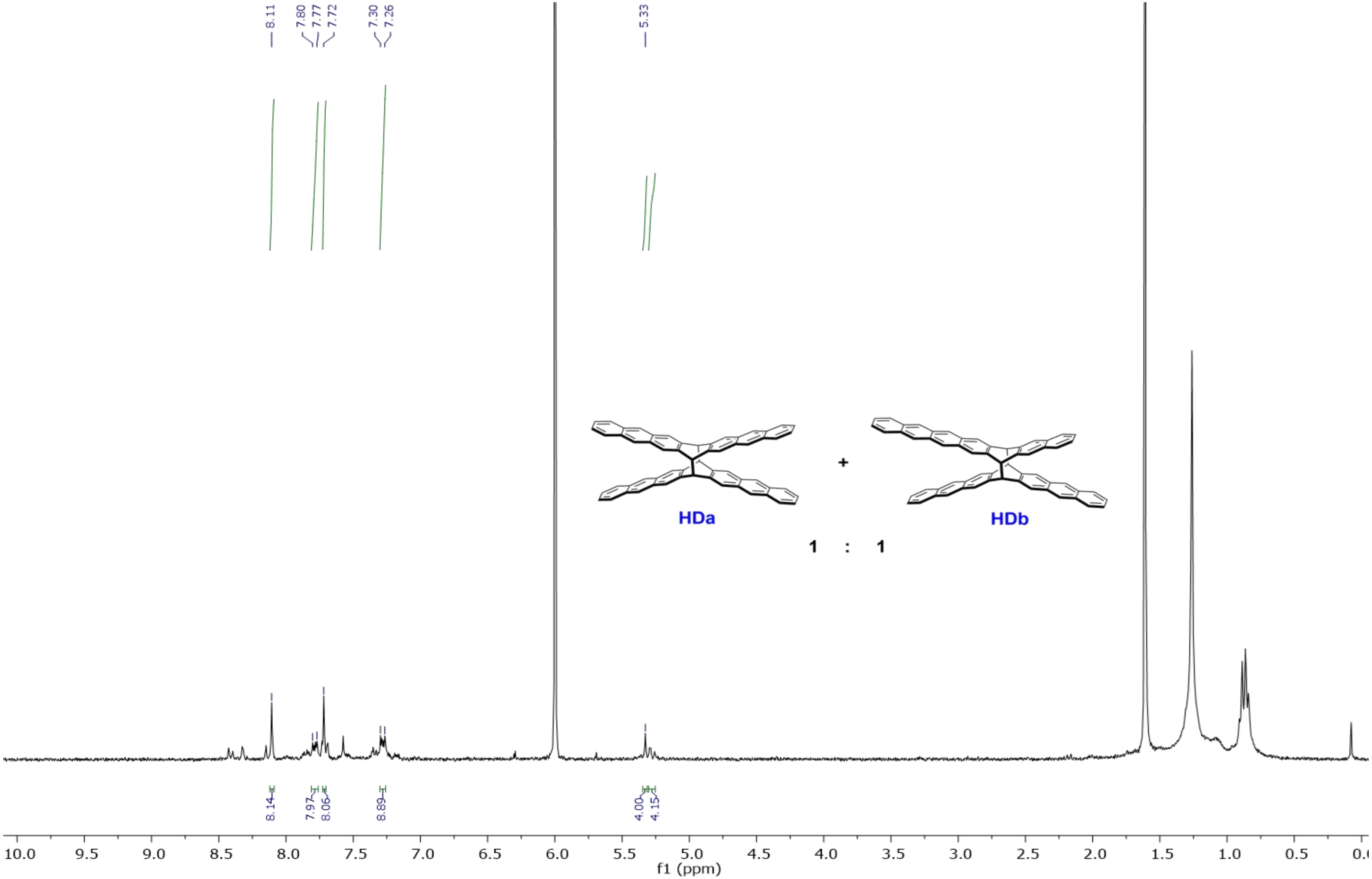
8.11
7.80
7.77
7.72
7.30
7.26
5.33
HDa
+
HDb
1 : 1
8.14
7.97
8.06
8.89
4.00
4.15
10.0 9.5 9.0 8.5 8.0 7.5 7.0 6.5 6.0 5.5 5.0 4.5 4.0 3.5 3.0 2.5 2.0 1.5 1.0 0.5
f1 (ppm)

Supplementary Figure 3 13C CPMAS spectra of transformations 7a/b → 1

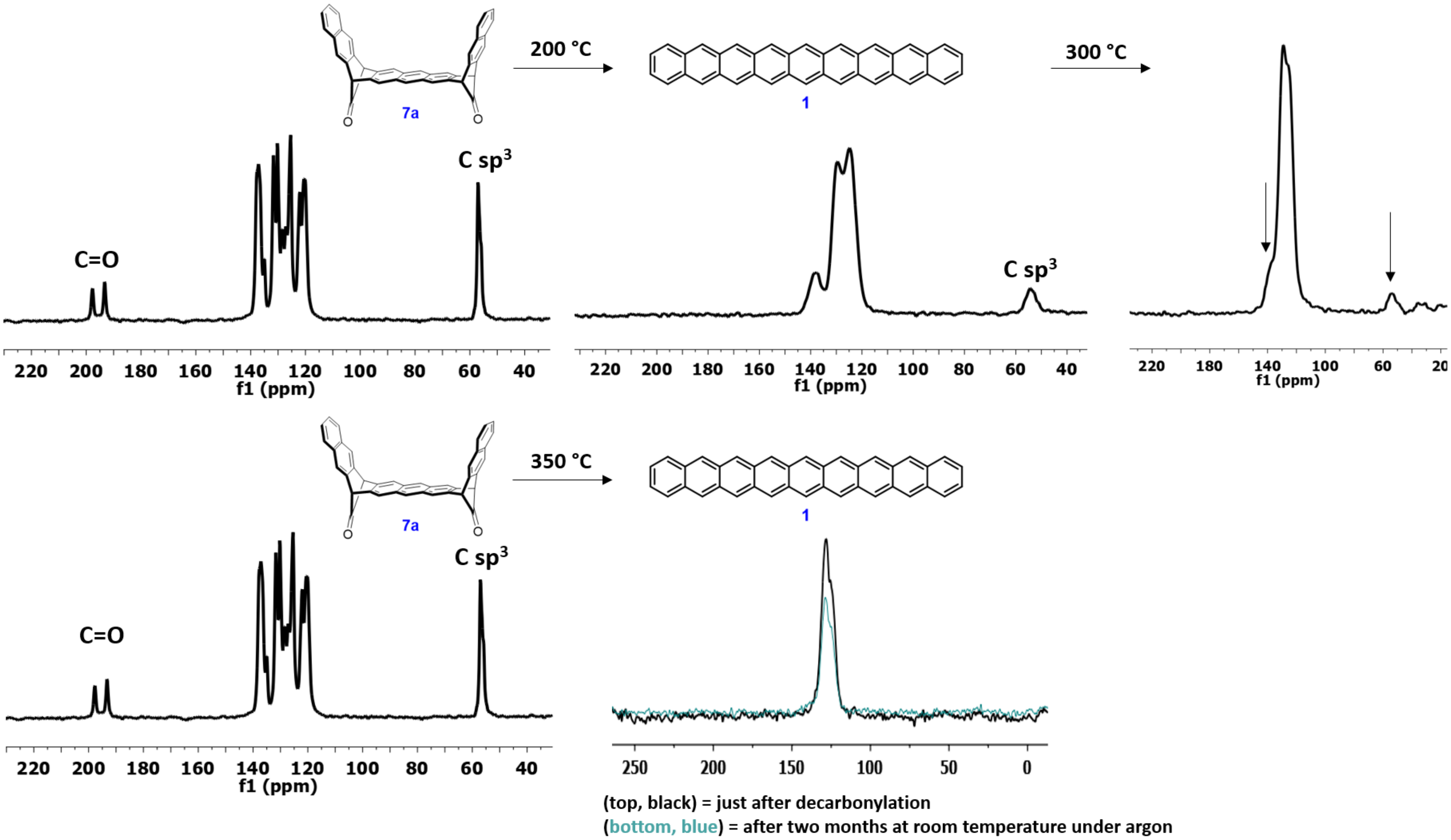

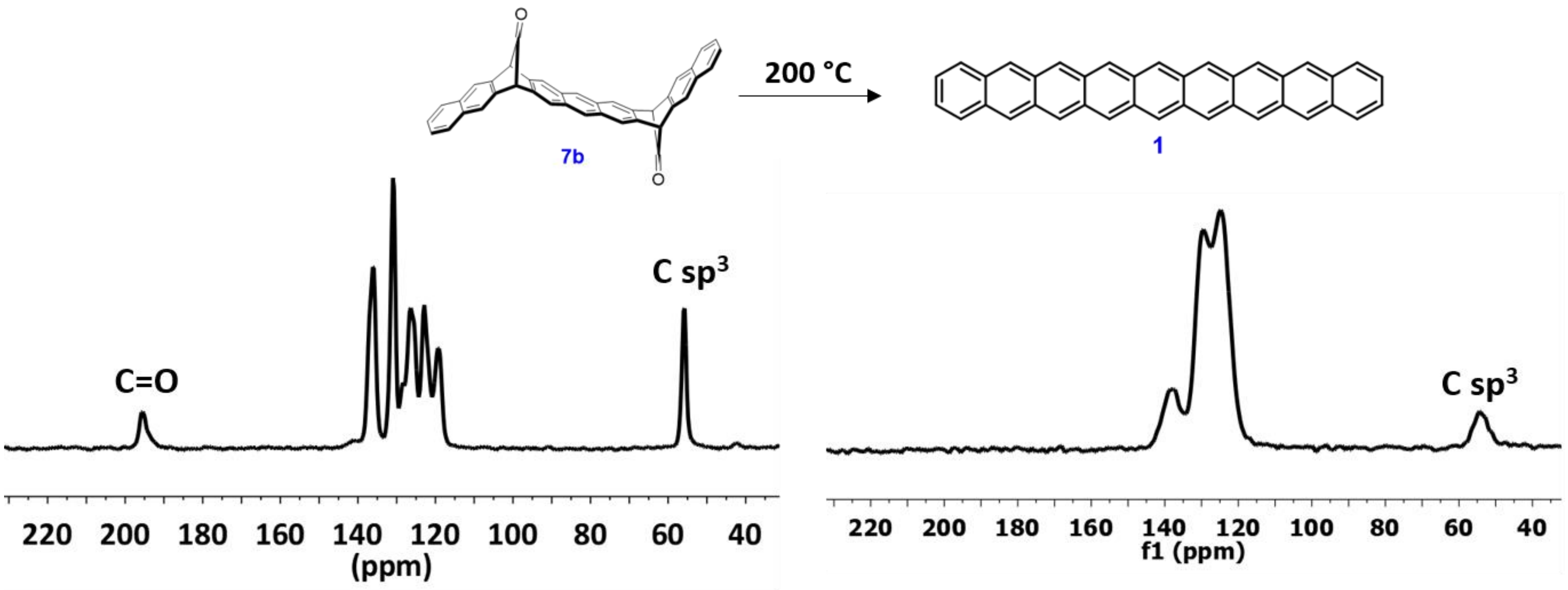

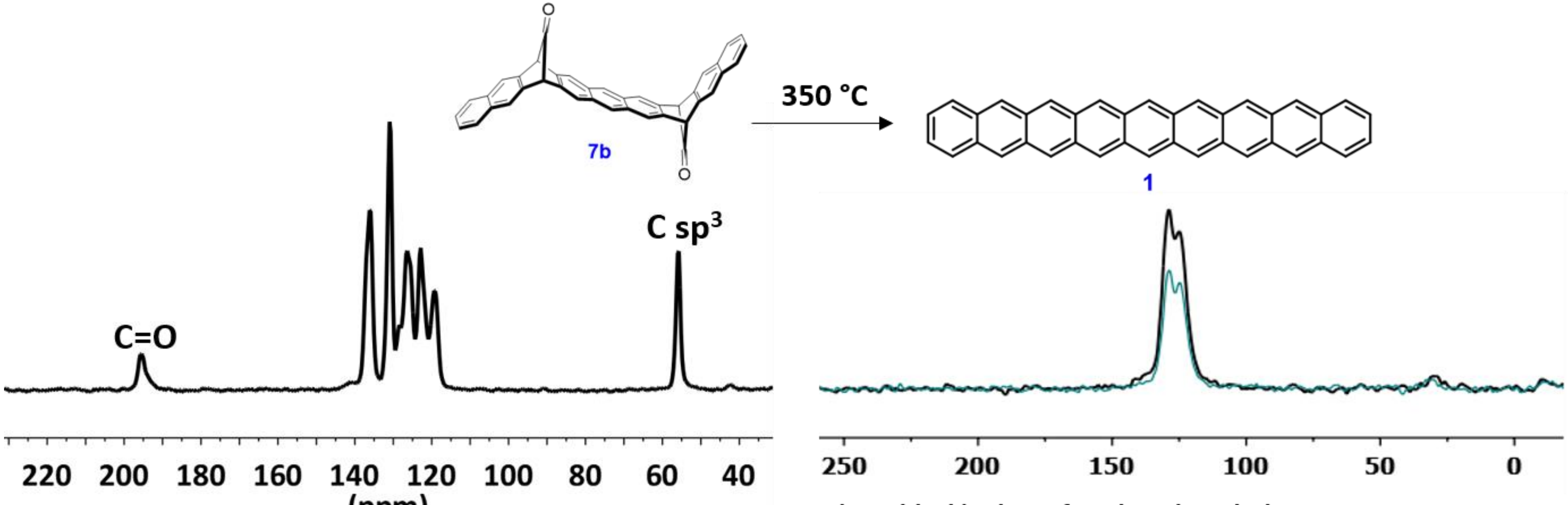

(top, black) = just after decarbonylation
(bottom, blue) = after two months at room temperature under argon

Supplementary Figure 4. IR spectra of transformations 7a/b → 1

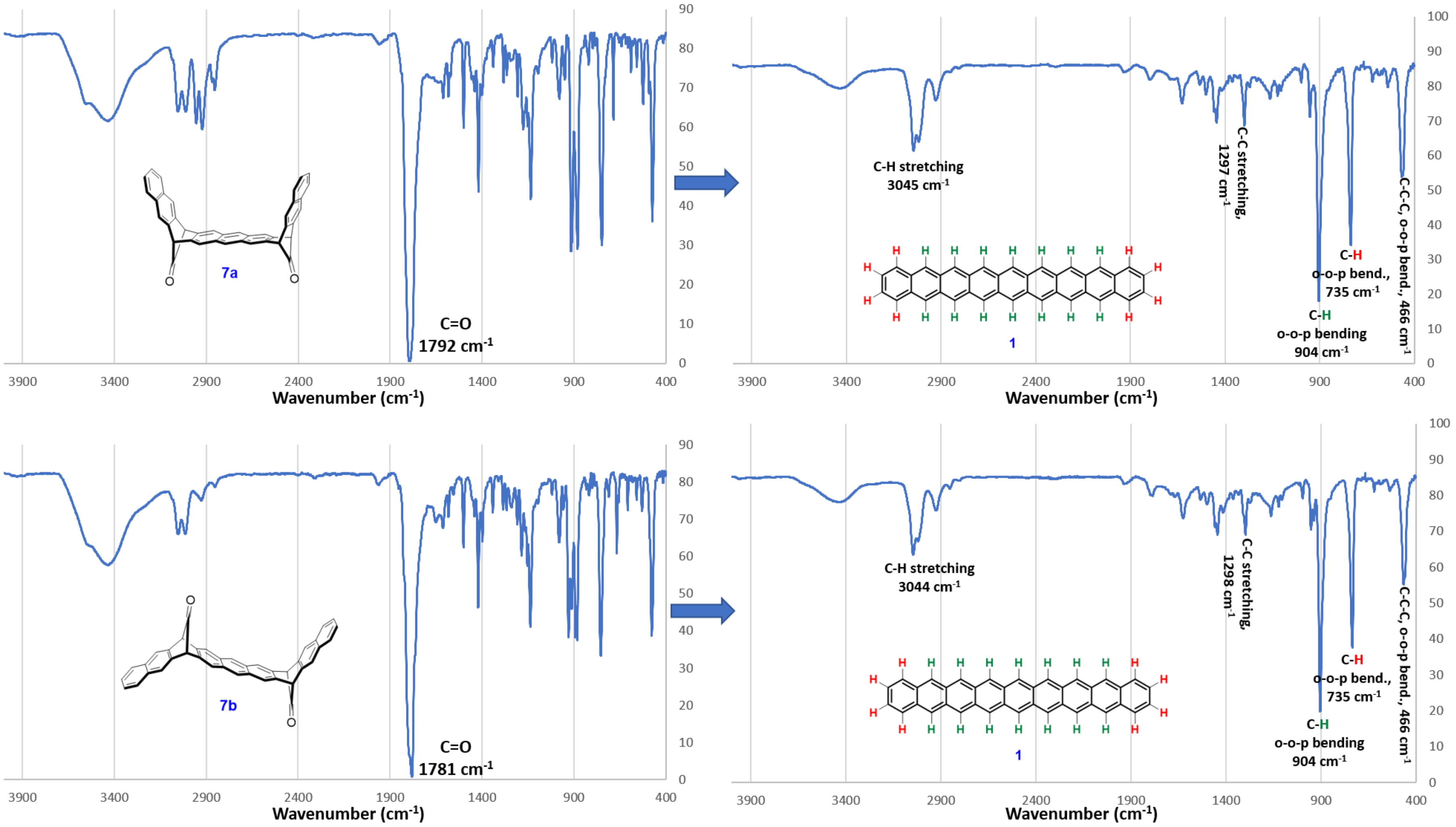

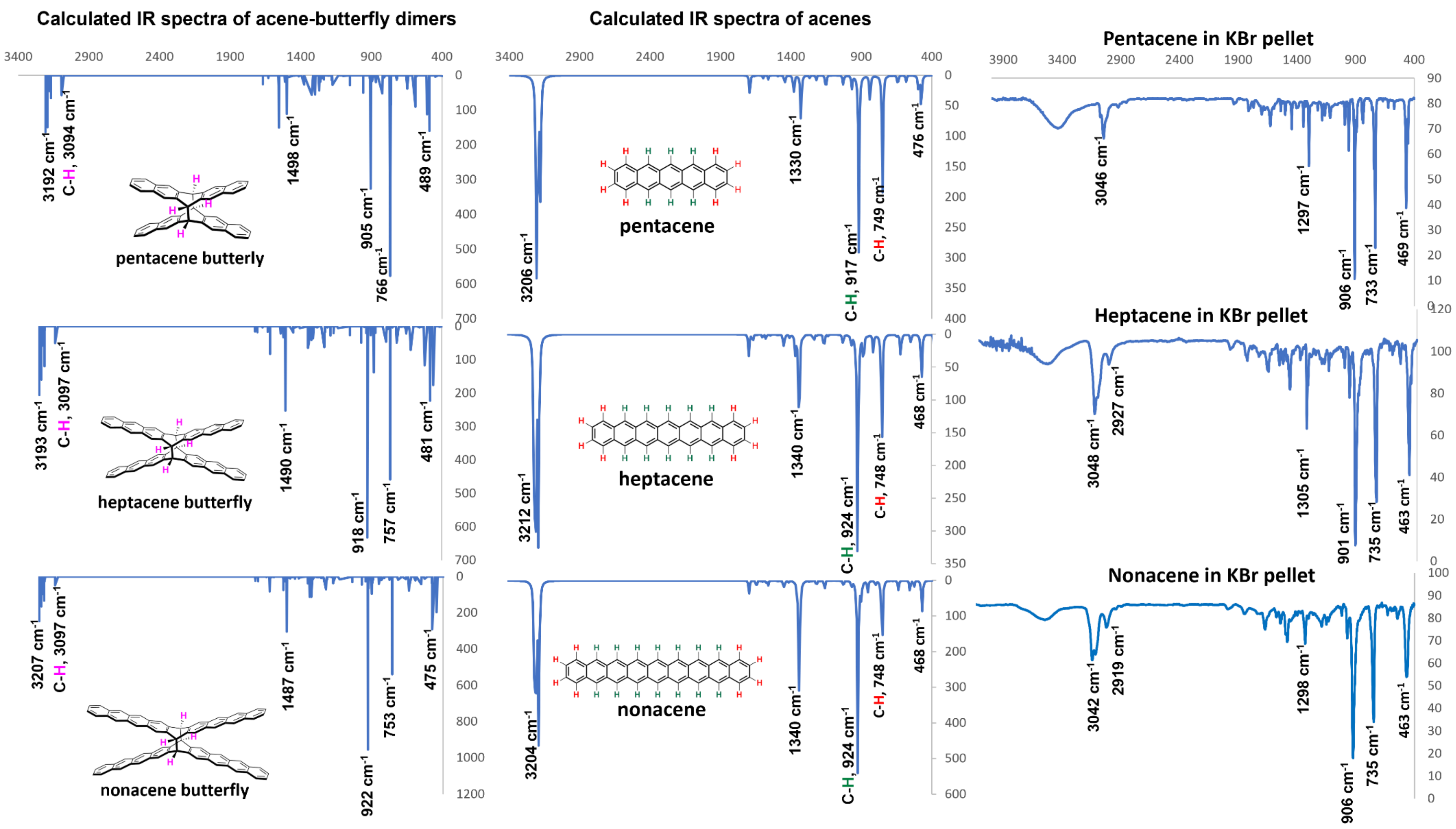
Calculated IR spectra of acene-butterfly dimers
Calculated IR spectra of acenes
Pentacene in KBr pellet
Heptacene in KBr pellet
Nonacene in KBr pellet
pentacene butterly
heptacene butterfly
nonacene butterfly
pentacene
heptacene
nonacene
3192 cm-1
C-H, 3094 cm-1
1498 cm-1
905 cm-1
766 cm-1
489 cm-1
3193 cm-1
C-H, 3097 cm-1
1490 cm-1
918 cm-1
757 cm-1
481 cm-1
3207 cm-1
C-H, 3097 cm-1
1487 cm-1
922 cm-1
753 cm-1
475 cm-1
3206 cm-1
1330 cm-1
C-H, 917 cm-1
C-H, 749 cm-1
476 cm-1
3212 cm-1
1340 cm-1
C-H, 924 cm-1
C-H, 748 cm-1
468 cm-1
3204 cm-1
1340 cm-1
C-H, 924 cm-1
C-H, 748 cm-1
468 cm-1
3046 cm-1
1297 cm-1
906 cm-1
733 cm-1
469 cm-1
3048 cm-1
2927 cm-1
1305 cm-1
901 cm-1
735 cm-1
463 cm-1
3042 cm-1
2919 cm-1
1298 cm-1
906 cm-1
735 cm-1
463 cm-1

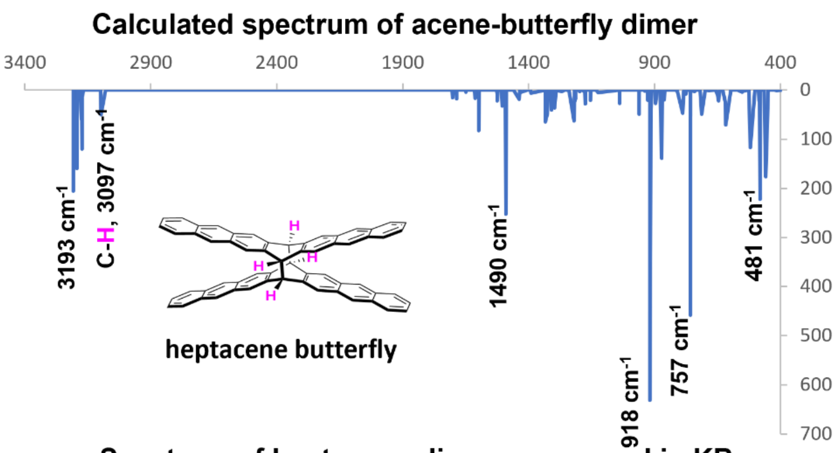
Calculated spectrum of acene-butterfly dimer
3400
2900
2400
1900
1400
900
400
0
100
200
300
400
500
600
700
3193 cm-1
C-H, 3097 cm-1
heptacene butterfly
1490 cm-1
918 cm-1
757 cm-1
481 cm-1

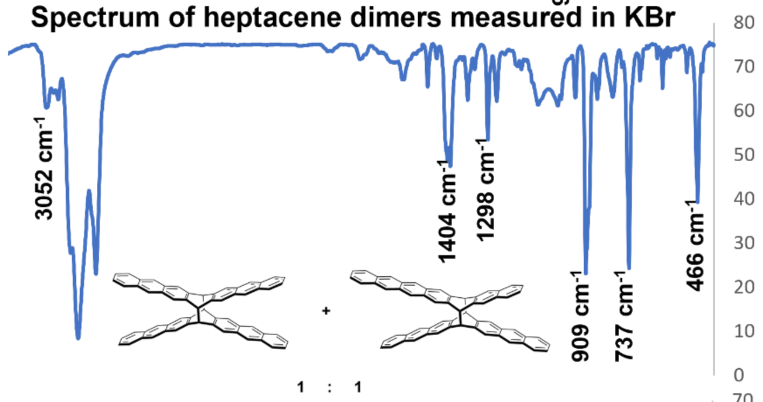
Spectrum of heptacene dimers measured in KBr
80
70
60
50
40
30
20
10
0
3052 cm-1
1404 cm-1
1298 cm-1
909 cm-1
737 cm-1
466 cm-1
+
1 : 1

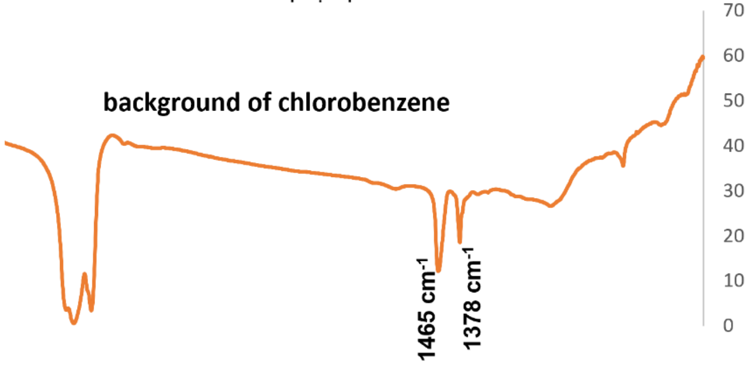
background of chlorobenzene
70
60
50
40
30
20
10
0
1465 cm-1
1378 cm-1

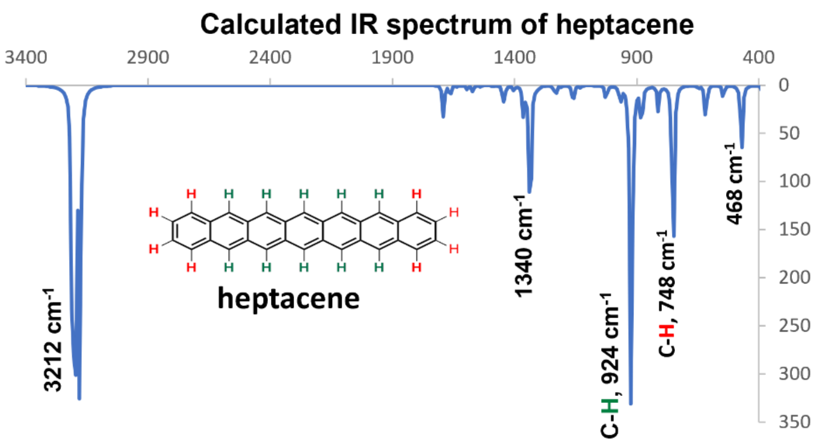
Calculated IR spectrum of heptacene
3400
2900
2400
1900
1400
900
400
0
50
100
150
200
250
300
350
3212 cm-1
heptacene
1340 cm-1
C-H, 924 cm-1
C-H, 748 cm-1
468 cm-1

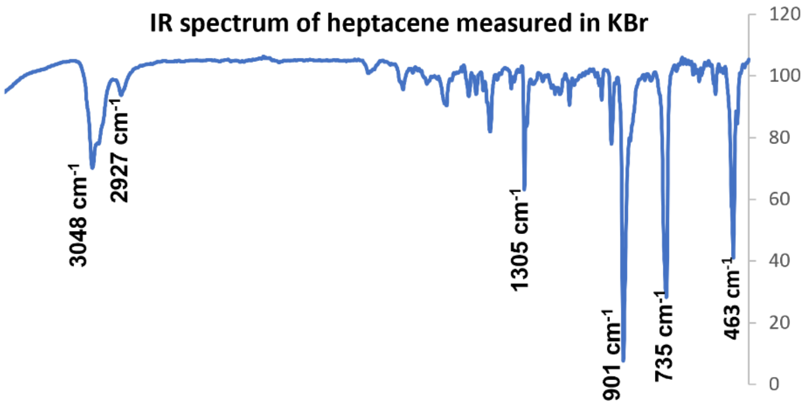
IR spectrum of heptacene measured in KBr
120
100
80
60
40
20
0
3048 cm-1
2927 cm-1
1305 cm-1
901 cm-1
735 cm-1
463 cm-1

Supplementary Figure 5. IR spectra measured in KBr

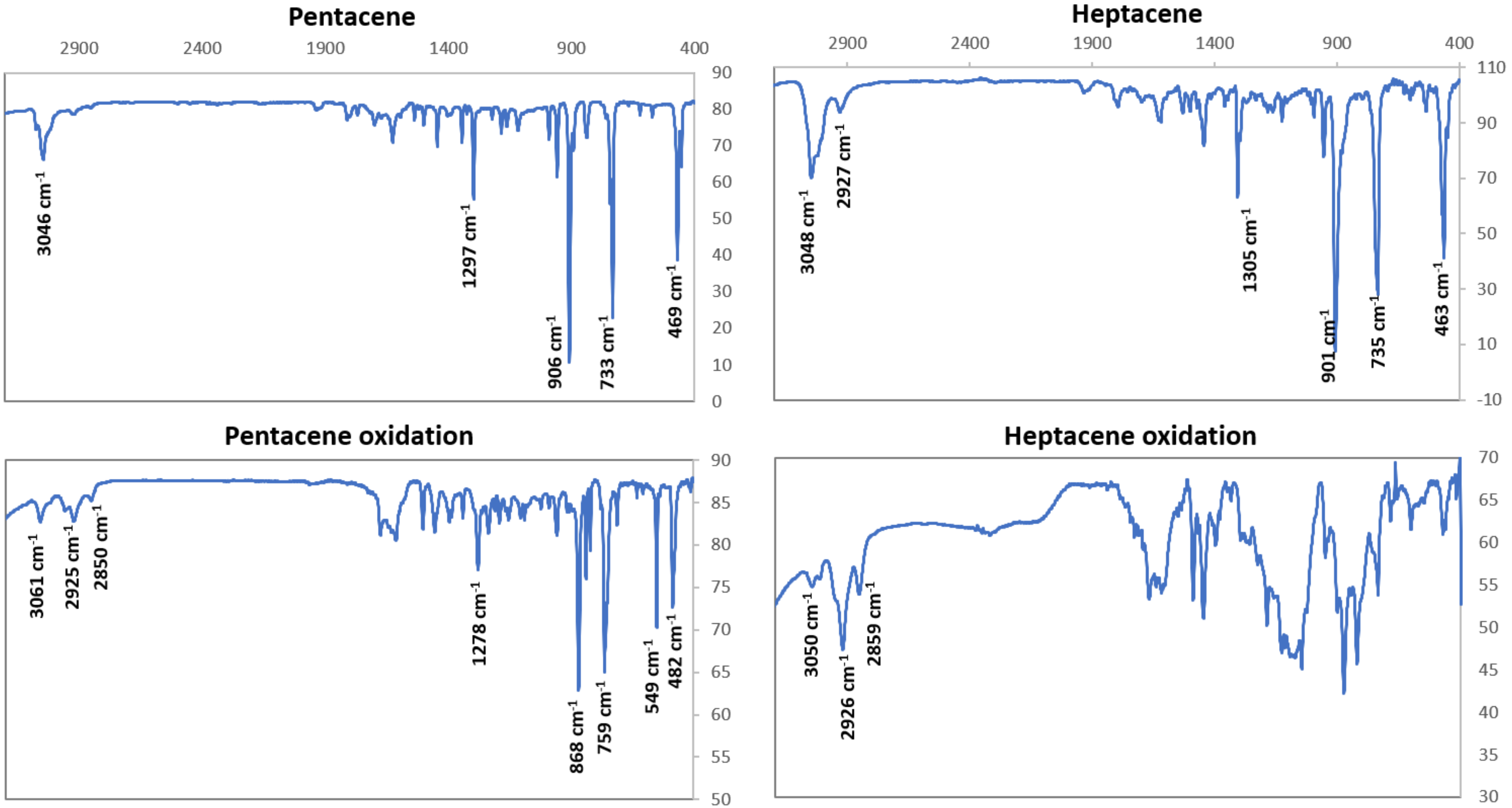

Nonacene generated in KBr pellet at 200°C

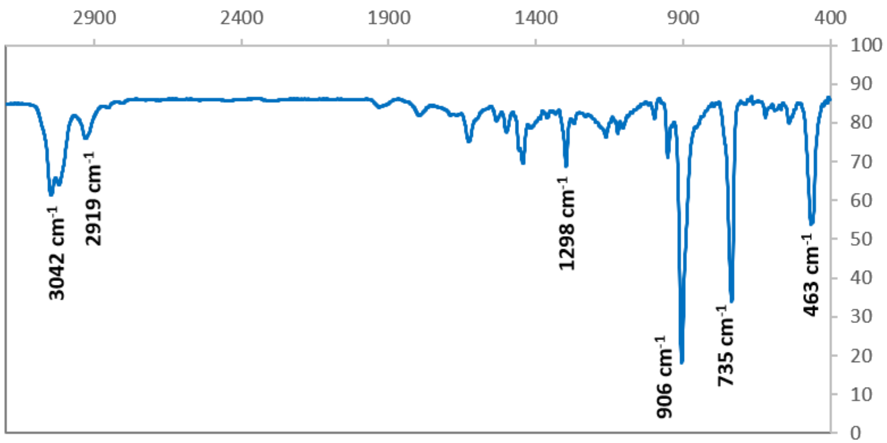

Nonacene generated in chlorobenzene at 200°C

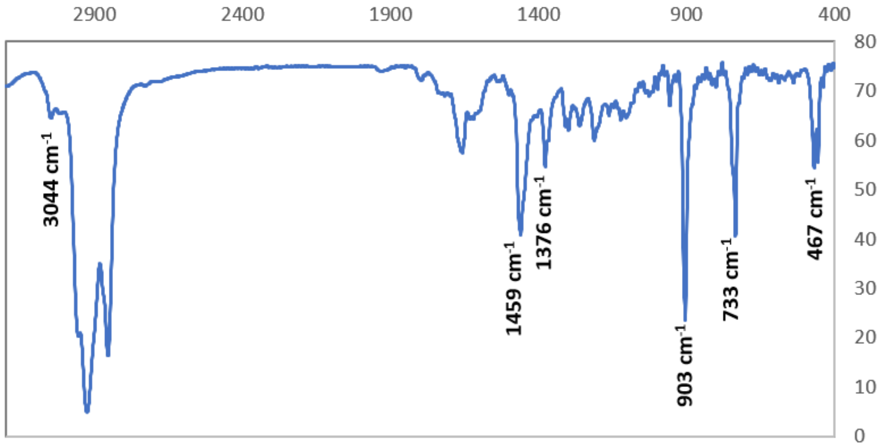

Nonacene generated in KBr pellet at 350°C

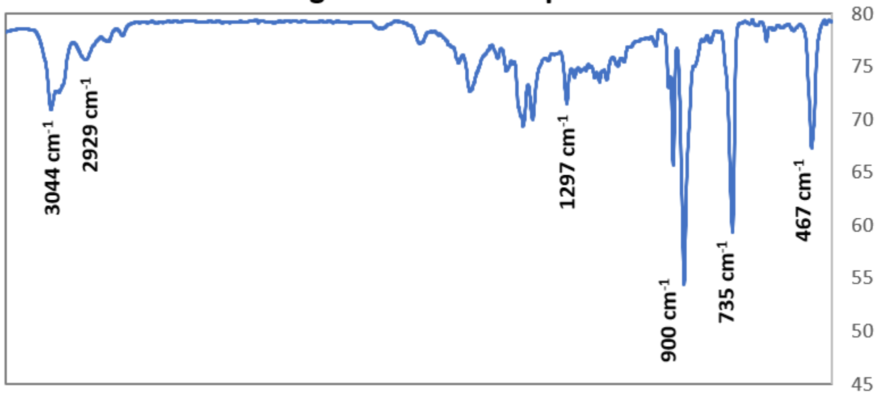

Background of chlorobenzene

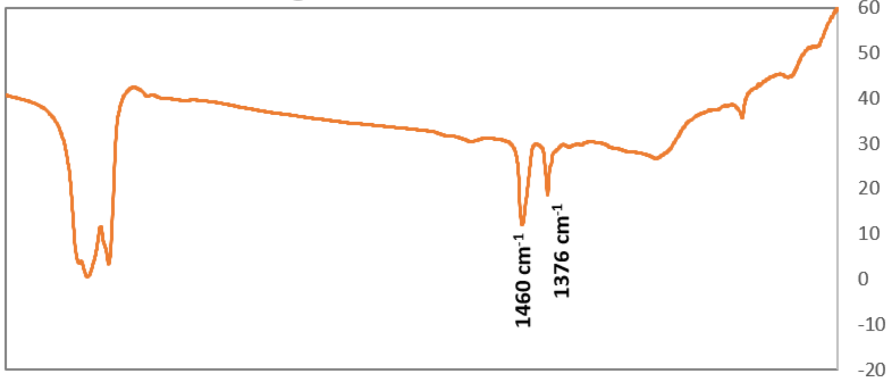

Supplementary Figure 6. Thermal Gravimetric Analysis of 7a/b

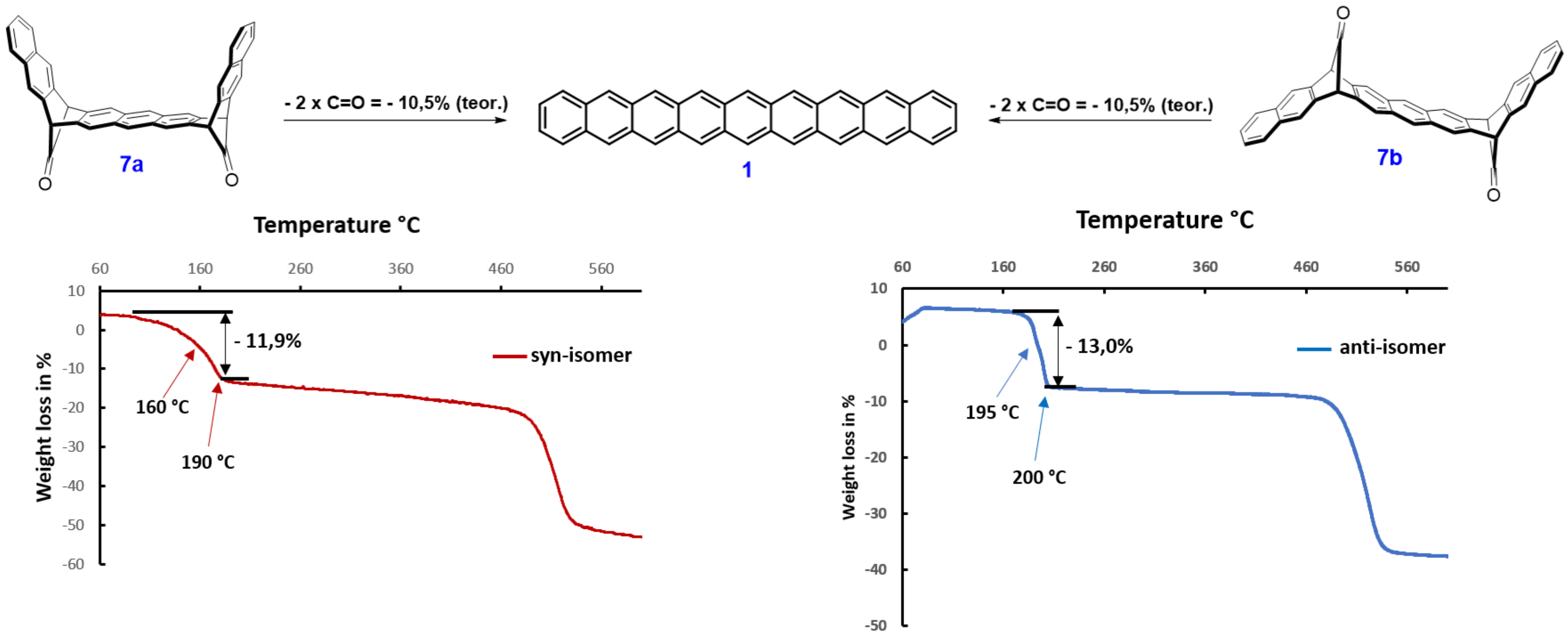

Supplementary Figure 7. MALDI MS Analysis of 1 generated in different conditions

MS MALDI spectrum of **1**, generated from **7a** in solid state form on ITO glass slide, measured without matrix.

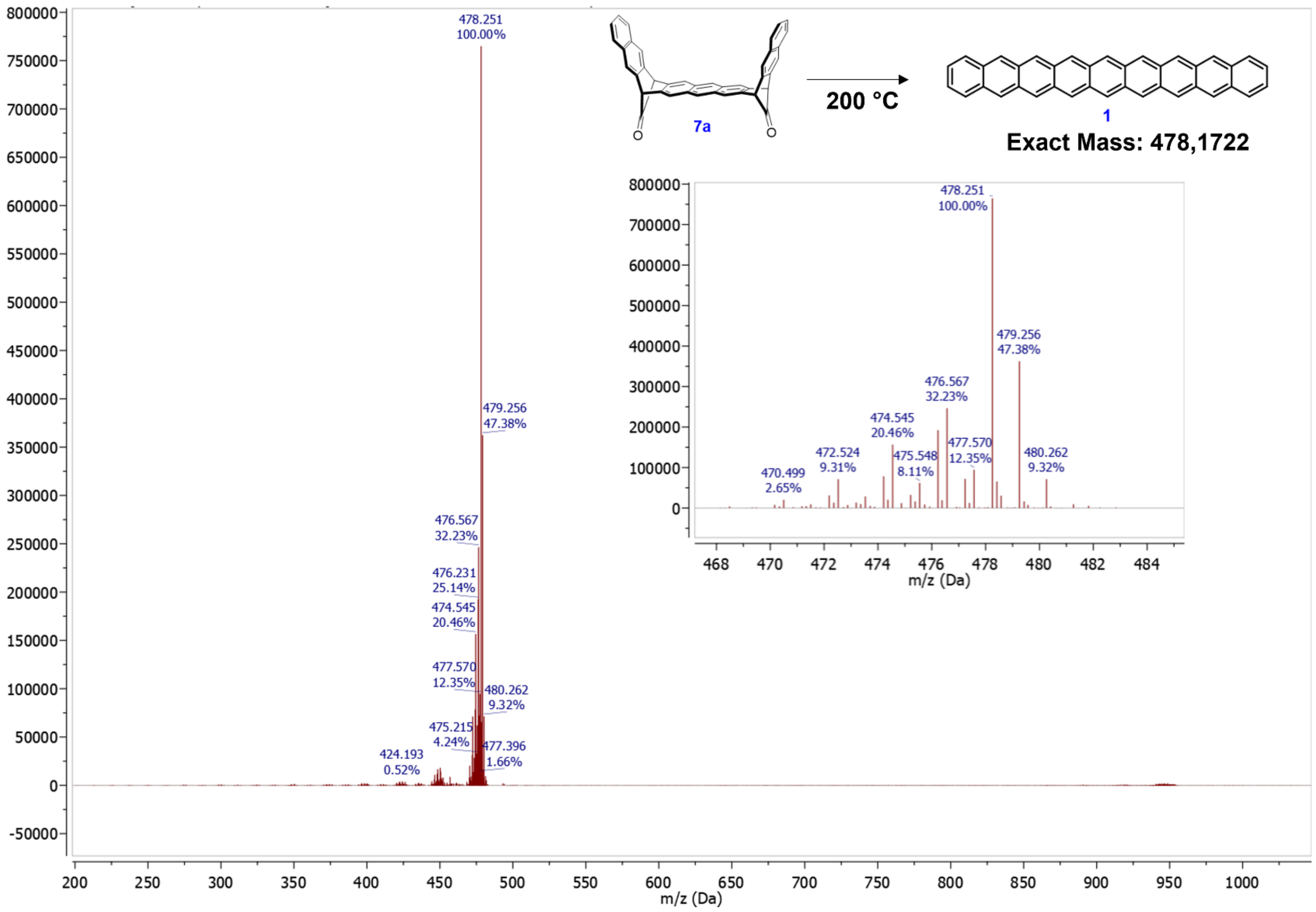

MS MALDI spectrum of **1**, generated from **7b** in solid state form on ITO glass slide, measured without matrix.

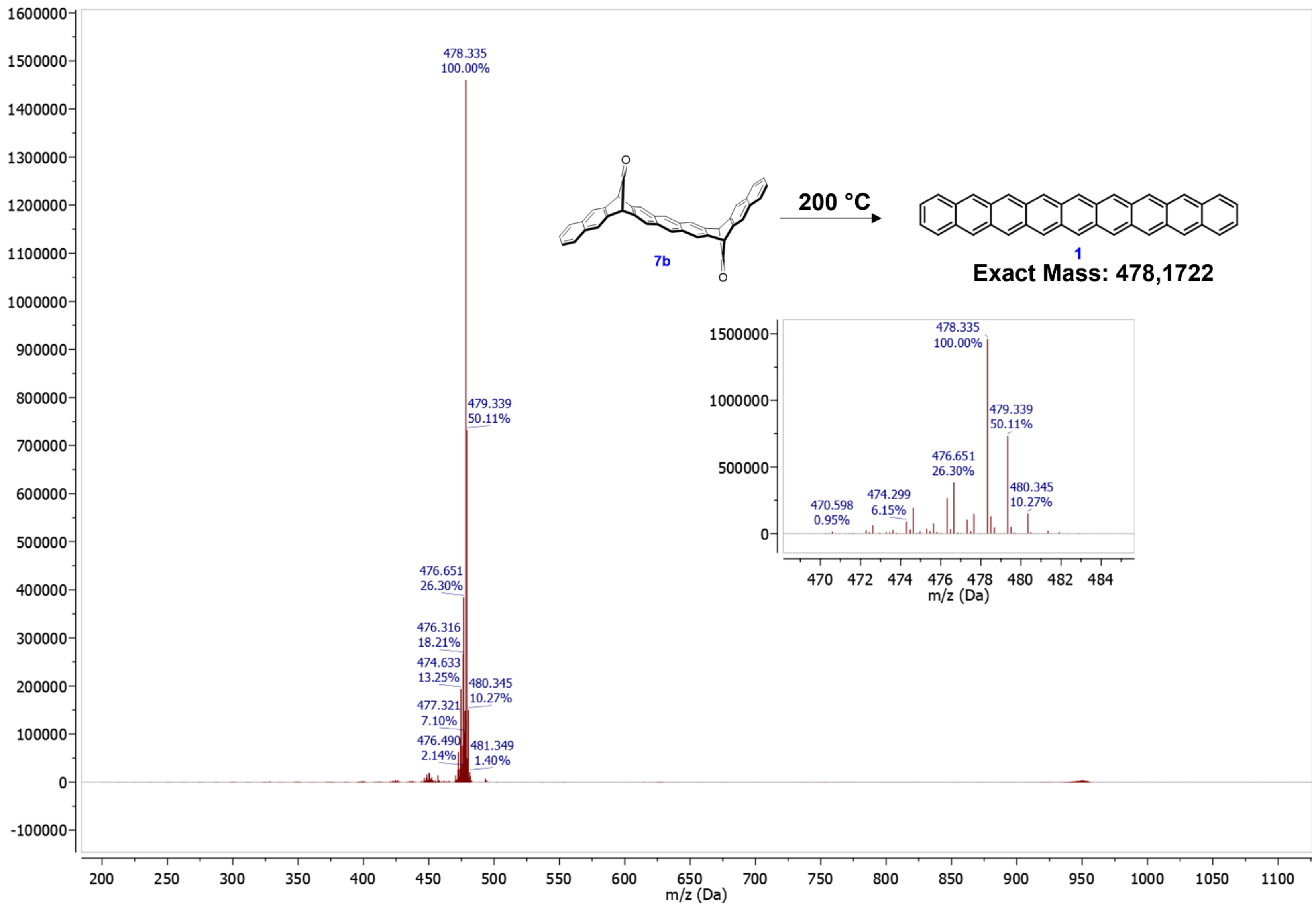

MS MALDI spectrum of **1**, generated from **7a** in chlorobenzene, measured without matrix.

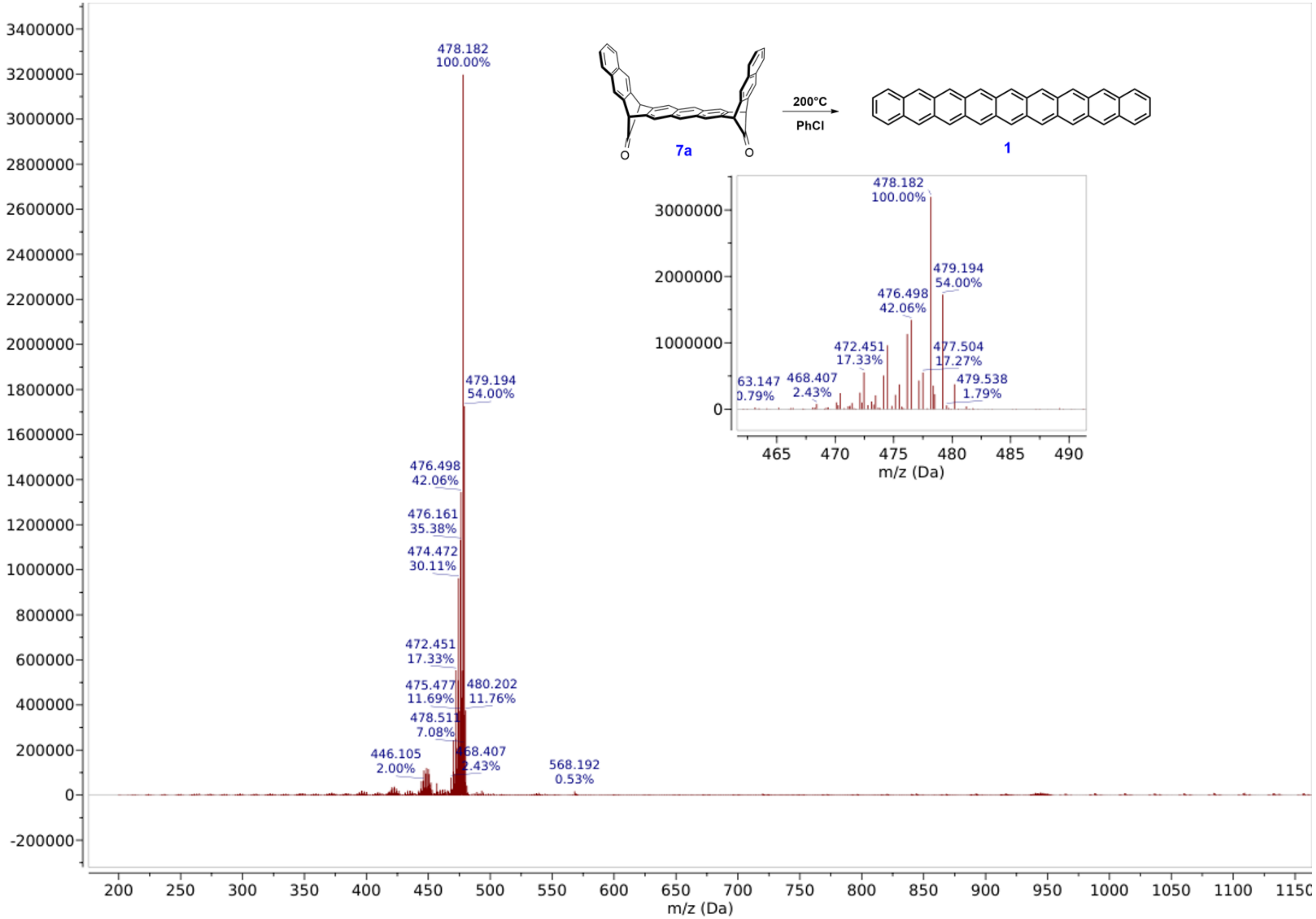

MS MALDI spectrum of **1**, generated from **7a** in chlorobenzene, measured with DHB as a matrix.

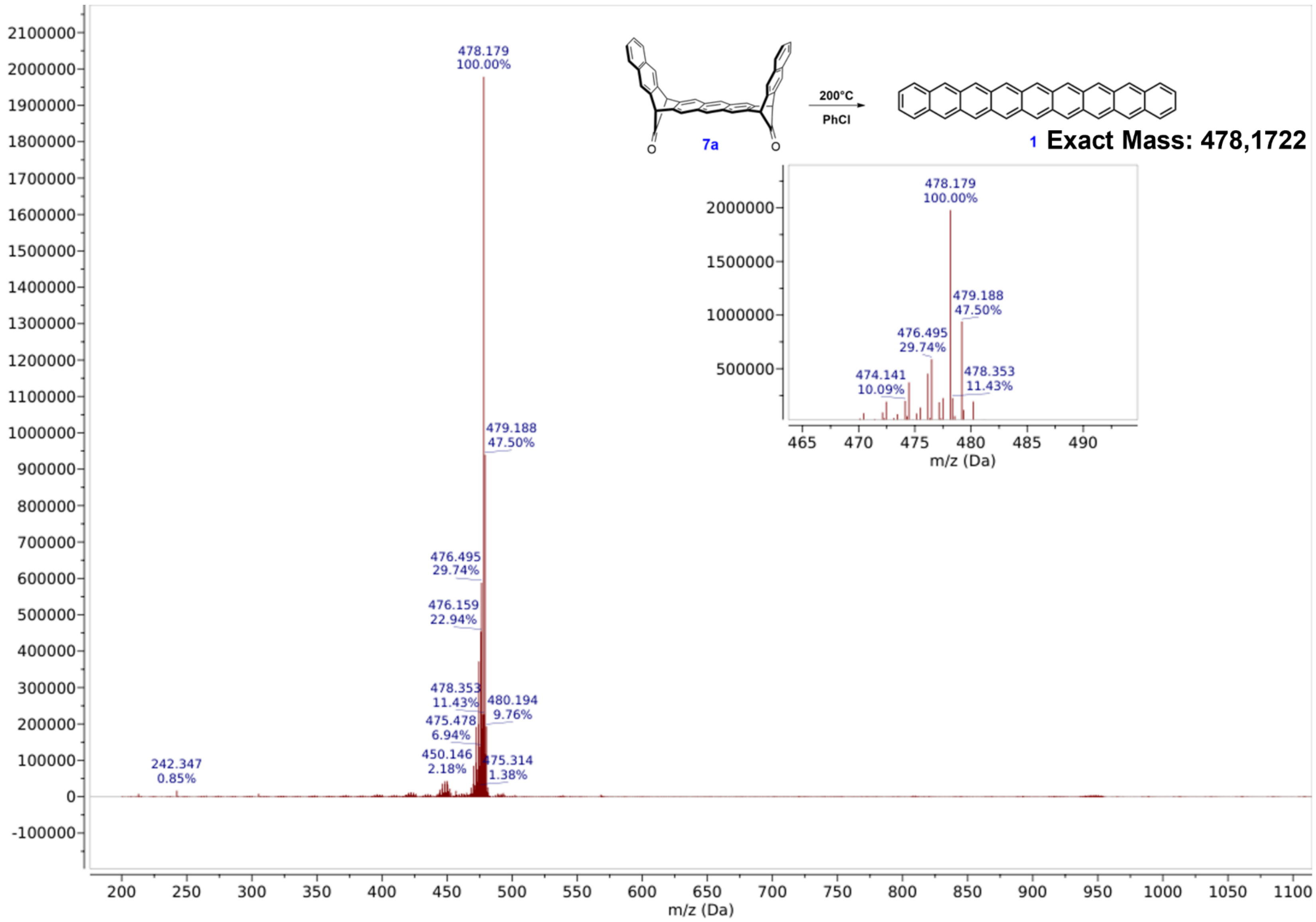

MS MALDI spectrum of **HP**.

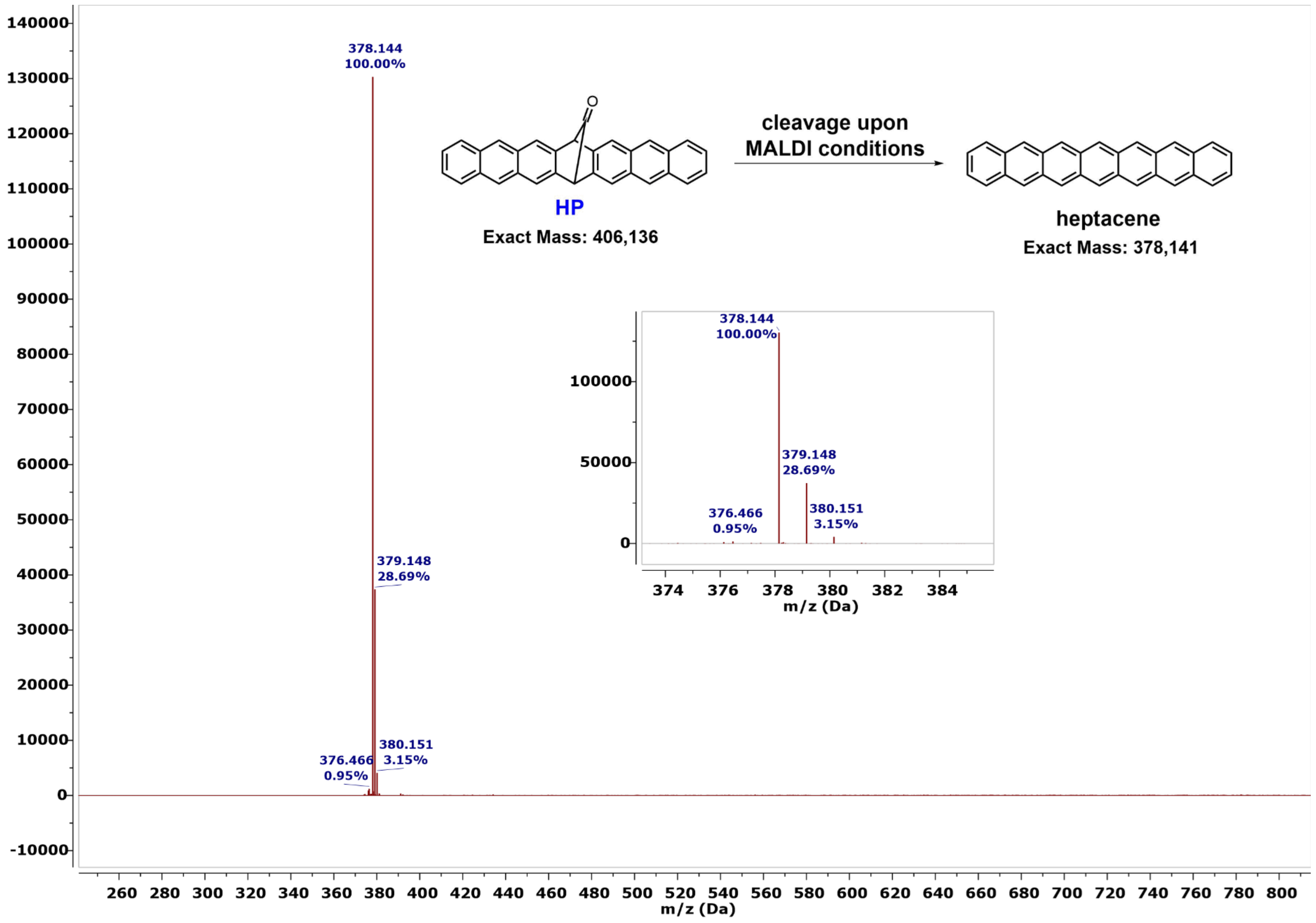

MS MALDI spectrum of **HP** measured using low energy laser.

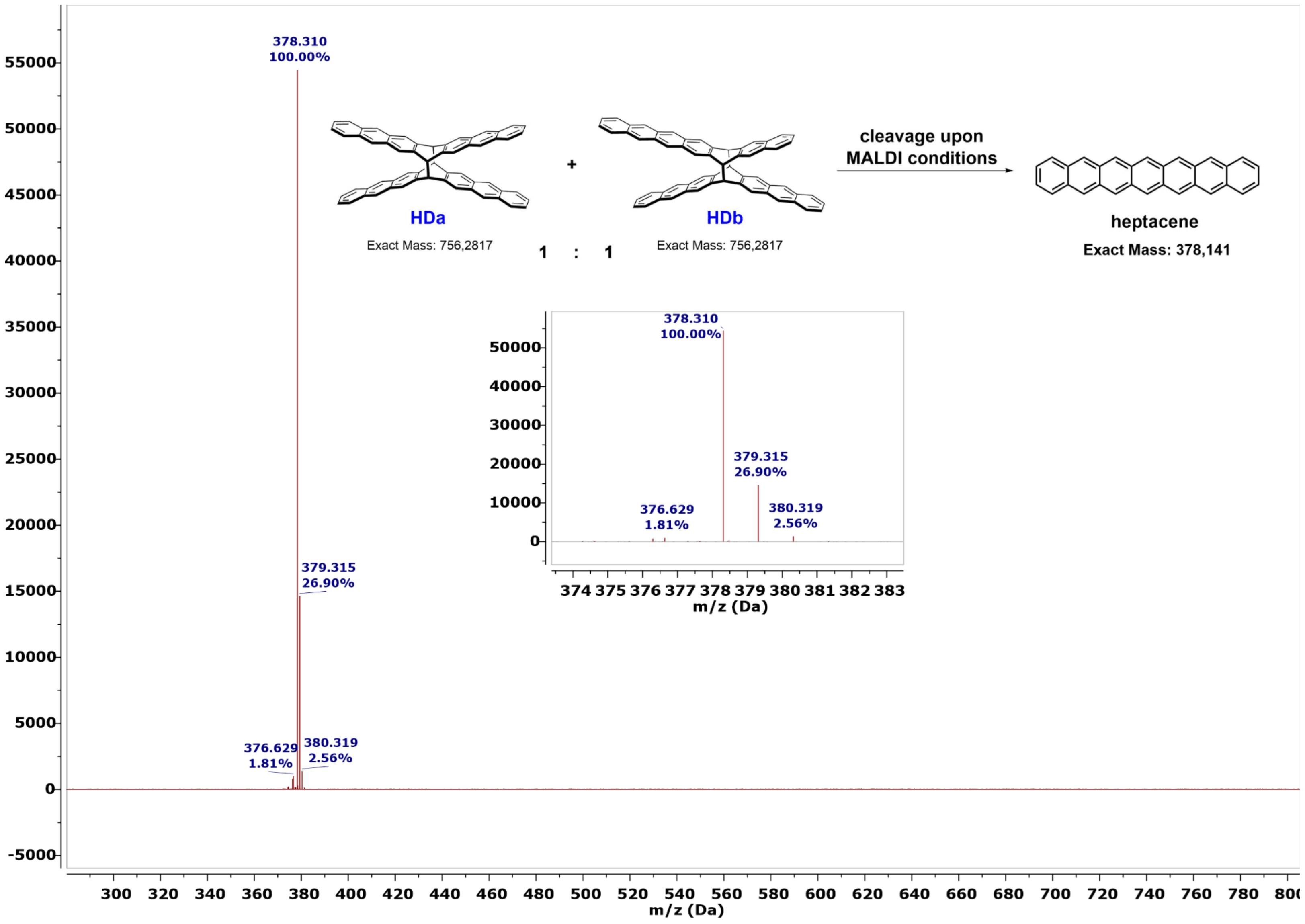

MS MALDI spectrum of **HP** measured using high energy laser.

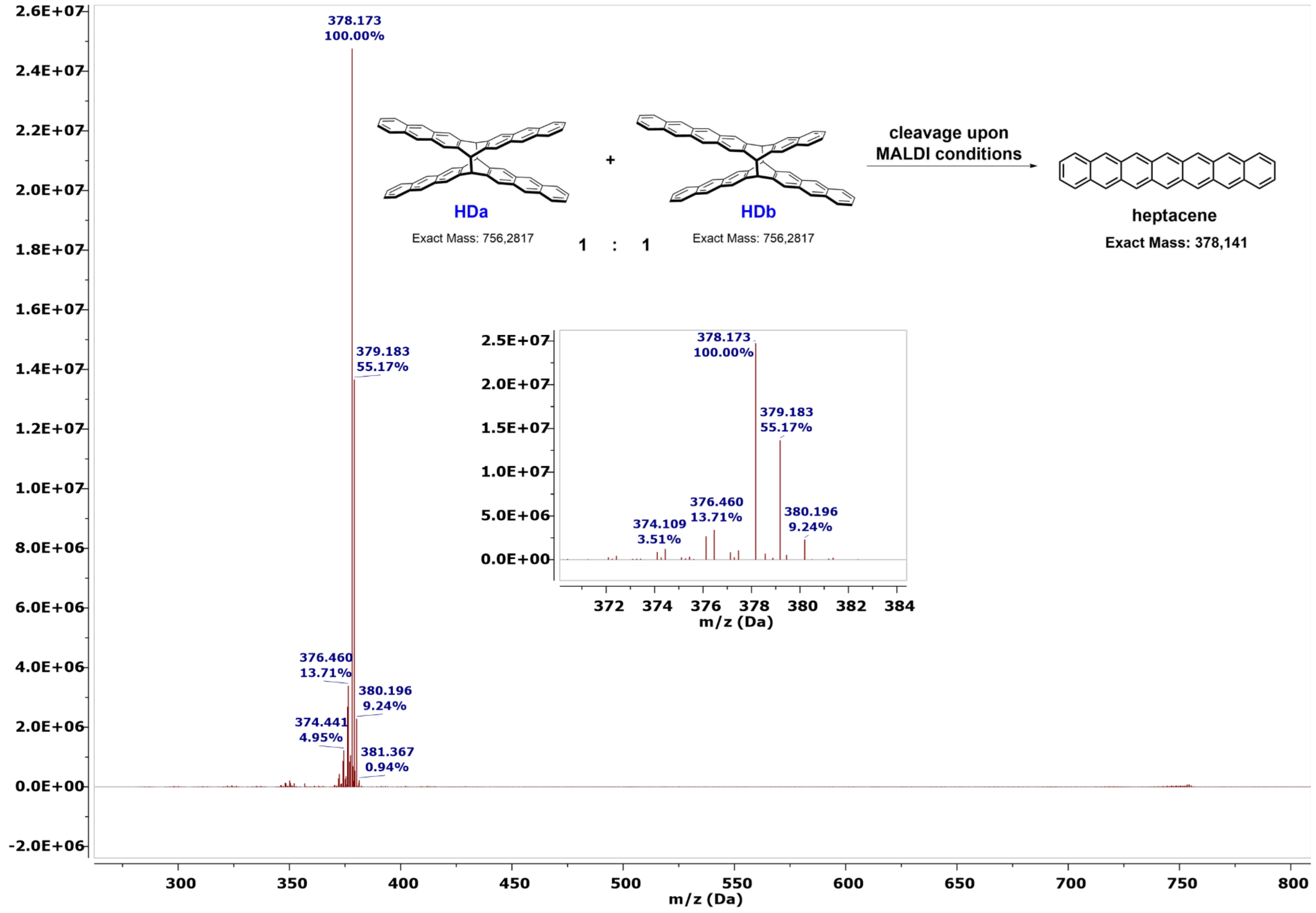

Supplementary Figure 8. Theoretical calculation of Diels Alder reaction

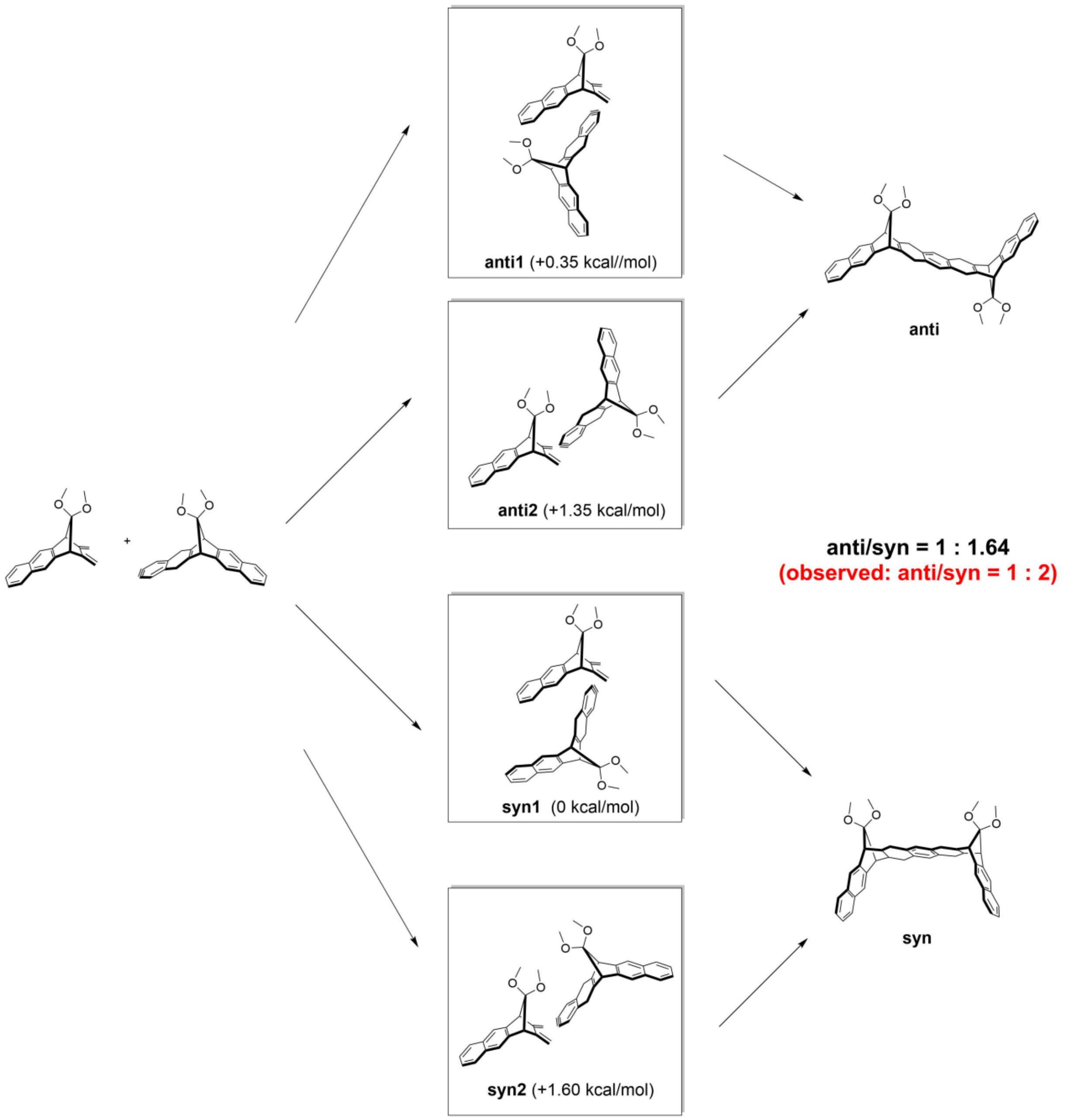

Gaussian 16, Revision A.03
M06-2X/6-31+G(d,p)//B3LYP/6-31+G(d,p)
Acetonirile (PCM method), 298.15 K

Supplementary Figure 9. X-ray analysis of 5b

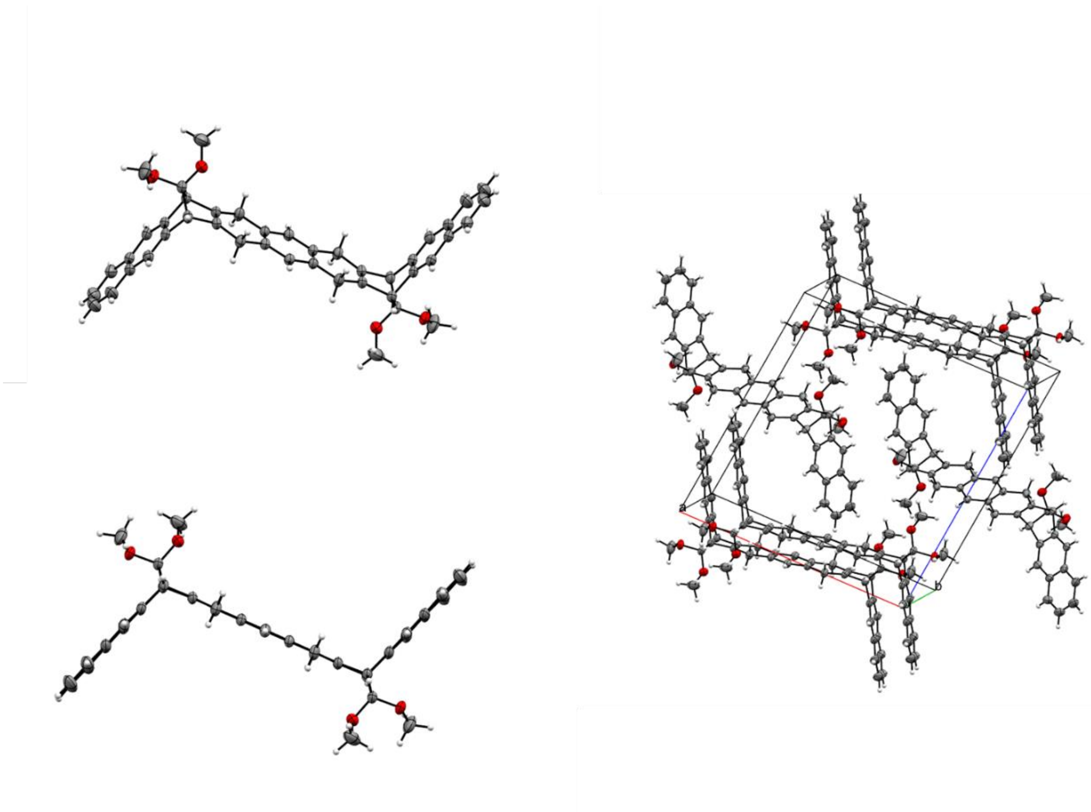

Single crystals of **5b** grown by slow evaporation of a mixture of hexane: EtOAc were selected in mother liquor from a flask and covered with perfluorated polyether oil on a microscope slide. An appropriate crystal was selected using a polarizing microscope, fixed on the tip of a MiTeGen© MicroMount, transferred to a goniometer head, and shock cooled by the crystalcooling device. Crystallographic data were collected at 193(2) K on a Bruker-AXS Kappa APEX II Quazar diffractometer equipped with a 30W air-cooled microfocus source using Mo K_{α} radiation (λ=0.71073 Å). Phi- and omega-scans were used. Space group was determined on the basis of systematic absences and intensity statistics. Semi-empirical absorption correction was employed.[ii] The structure was solved using an intrinsic phasing method (SHELXT),[5] and refined using the least-squares method on F^2. All non-H atoms were refined with anisotropic displacement parameters. Hydrogen atoms were refined isotropically at calculated positions using a riding model with their isotropic displacement parameters constrained to be equal to 1.5 times the equivalent isotropic displacement parameters of their pivot atoms for terminal sp^3 carbon and 1.2 times for all other carbon atoms.

The solvent molecule (ethyl acetate on a special position) was disordered over two positions, for which occupancies were refined. Several restraints (SAME, SIMU, DELU) were applied to refine the molecule and to avoid the collapse of the structure during the least-squares refinement by the large anisotropic displacement parameters. A bond length was restrained with DFIX to suitable target values.

CCDC-2071402 contains the supplementary crystallographic data for this paper. These data can be obtained free of charge from The Cambridge Crystallographic Data Centre via https://www.ccdc.cam.ac.uk/structures/.

Selected data for 5b: $C_{44}H_{38}O_4$, $C_4H_8O_2$ *M* = 718.85, Monoclinic, space group *P*2_1*/n*, *a* = 16.0184(9) Å, *b* = 7.0568(5) Å, *c* = 16.9607(11) Å, β = 99.492(2)°, *V* = 1891.0(2) $Å^3$, *Z* = 2, crystal size 0.24 x 0.22 x 0.15 mm^3, 49831 reflections collected (4682 independent, R*int* = 0.0659), 328 parameters, 193 restraints, *R*1 [I>2σ(I)] = 0.0499, *wR*2 [all data] = 0.1425, largest diff. peak and hole: 0.268 and –0.193 $eÅ^{-3}$.

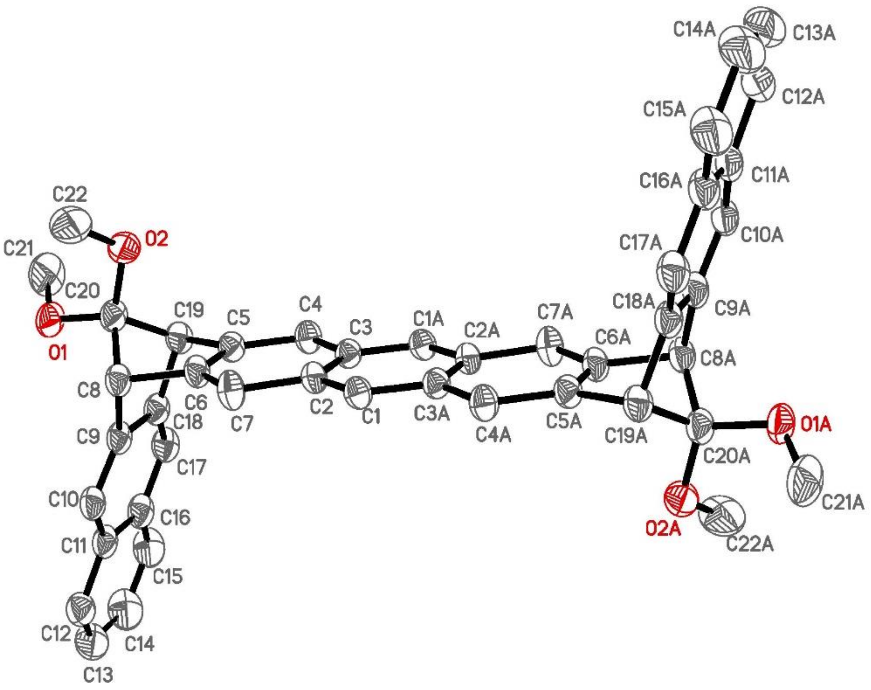

Supplementary Figure 10. Molecular structure of **5b**. Thermal ellipsoids represent 50% probability level. Hydrogen atoms and solvent molecule were omitted for clarity.

Supplementary Table 1. Crystal data and structure refinement for 5b.

Identification code	AJ189_a
Empirical formula	C48 H46 O6
Formula weight	718.85
Temperature	193(2) K
Wavelength	0.71073 Å
Crystal system	Monoclinic
Space group	$P2_1/n$

Unit cell dimensions	a = 16.0184(9) Å	α= 90°.
	b = 7.0568(5) Å	β= 99.492(2)°.
	c = 16.9607(11) Å	γ = 90°.
Volume	1891.0(2) $Å^3$	
Z	2	
Density (calculated)	1.262 Mg/m^3	
Absorption coefficient	0.082 mm^{-1}	
F(000)	764	
Crystal size	0.240 x 0.220 x 0.150 mm^3	
Theta range for data collection	3.162 to 28.278°.	
Index ranges	-21<=h<=21, -9<=k<=9, -22<=l<=22	
Reflections collected	49831	
Independent reflections	4682 [R(int) = 0.0659]	
Completeness to theta = 25.242°	99.6 %	
Absorption correction	Semi-empirical from equivalents	
Max. and min. transmission	0.7457 and 0.6994	
Refinement method	Full-matrix least-squares on F^2	
Data / restraints / parameters	4682 / 193 / 328	
Goodness-of-fit on F^2	1.022	
Final R indices [I>2sigma(I)]	R1 = 0.0499, wR2 = 0.1236	
R indices (all data)	R1 = 0.0834, wR2 = 0.1425	
Extinction coefficient	n/a	
Largest diff. peak and hole	0.268 and -0.193 e.$Å^{-3}$	

Supplementary Table 2. Atomic coordinates (x 104) and equivalent isotropic displacement parameters (Å2x 103) for 5b. U(eq) is defined as one third of the trace of the orthogonalized U^{ij} tensor.

	x	y	z	U(eq)
O(1)	3955(1)	9047(2)	4269(1)	42(1)
O(2)	2558(1)	9149(2)	3651(1)	42(1)
C(1)	186(1)	3395(2)	4614(1)	29(1)
C(2)	792(1)	4818(2)	4749(1)	29(1)
C(3)	604(1)	6461(2)	5146(1)	28(1)
C(4)	1216(1)	8102(2)	5317(1)	33(1)
C(5)	2014(1)	7758(2)	5007(1)	31(1)
C(6)	2206(1)	6158(2)	4652(1)	31(1)
C(7)	1638(1)	4524(3)	4475(1)	37(1)
C(8)	3119(1)	6335(3)	4504(1)	33(1)
C(9)	3665(1)	6383(2)	5332(1)	32(1)
C(10)	4243(1)	5169(3)	5725(1)	35(1)
C(11)	4636(1)	5601(3)	6522(1)	38(1)
C(12)	5225(1)	4359(3)	6966(1)	46(1)
C(13)	5609(1)	4809(4)	7724(1)	58(1)
C(14)	5429(1)	6521(4)	8071(1)	63(1)
C(15)	4860(1)	7757(4)	7663(1)	53(1)
C(16)	4441(1)	7334(3)	6883(1)	40(1)
C(17)	3843(1)	8598(3)	6443(1)	38(1)
C(18)	3458(1)	8109(2)	5698(1)	33(1)
C(19)	2784(1)	9071(2)	5096(1)	33(1)
C(20)	3122(1)	8499(3)	4315(1)	36(1)
C(21)	4074(1)	11042(3)	4253(2)	64(1)
C(22)	2713(2)	8396(4)	2908(1)	63(1)
C(23)	2671(12)	3550(30)	7124(13)	94(4)
C(24)	2206(11)	1910(20)	6874(10)	70(3)
O(3)	2609(12)	990(20)	7575(11)	73(2)
C(25)	2322(16)	-630(30)	7805(11)	64(3)
O(4)	1801(5)	-1423(12)	7279(5)	81(3)
C(26)	2751(14)	-1700(20)	8488(10)	58(3)
C(23')	2741(14)	-1150(20)	8368(11)	57(3)
C(24')	2289(15)	-590(30)	7596(10)	65(3)

O(3')	2544(12)	1400(20)	7548(11)	68(2)
C(25')	2374(10)	2490(20)	6931(10)	68(2)
O(4')	1957(5)	1619(12)	6338(5)	82(3)
C(26')	2322(14)	4610(20)	6822(12)	94(4)

Supplementary Table 3. Bond lengths [Å] and angles [°] for 5b.

O(1)-C(20)	1.4039(18)
O(1)-C(21)	1.422(2)
O(2)-C(20)	1.400(2)
O(2)-C(22)	1.425(2)
C(1)-C(2)	1.389(2)
C(1)-C(3)#1	1.395(2)
C(1)-H(1)	0.9500
C(2)-C(3)	1.398(2)
C(2)-C(7)	1.519(2)
C(3)-C(4)	1.513(2)
C(4)-C(5)	1.481(2)
C(4)-H(4A)	0.9900
C(4)-H(4B)	0.9900
C(5)-C(6)	1.339(2)
C(5)-C(19)	1.531(2)
C(6)-C(7)	1.469(2)
C(6)-C(8)	1.530(2)
C(7)-H(7A)	0.9900
C(7)-H(7B)	0.9900
C(8)-C(9)	1.529(2)
C(8)-C(20)	1.560(3)
C(8)-H(8)	1.0000
C(9)-C(10)	1.353(2)
C(9)-C(18)	1.430(2)
C(10)-C(11)	1.427(2)
C(10)-H(10)	0.9500
C(11)-C(12)	1.412(3)
C(11)-C(16)	1.425(3)
C(12)-C(13)	1.367(3)
C(12)-H(12)	0.9500
C(13)-C(14)	1.395(3)
C(13)-H(13)	0.9500
C(14)-C(15)	1.365(3)
C(14)-H(14)	0.9500
C(15)-C(16)	1.414(2)
C(15)-H(15)	0.9500

C(16)-C(17)	1.427(3)
C(17)-C(18)	1.357(2)
C(17)-H(17)	0.9500
C(18)-C(19)	1.518(2)
C(19)-C(20)	1.563(2)
C(19)-H(19)	1.0000
C(21)-H(21A)	0.9800
C(21)-H(21B)	0.9800
C(21)-H(21C)	0.9800
C(22)-H(22A)	0.9800
C(22)-H(22B)	0.9800
C(22)-H(22C)	0.9800
C(23)-C(24)	1.41(2)
C(23)-H(23A)	0.9800
C(23)-H(23B)	0.9800
C(23)-H(23C)	0.9800
C(24)-O(3)	1.414(9)
C(24)-H(24A)	0.9900
C(24)-H(24B)	0.9900
O(3)-C(25)	1.313(19)
C(25)-O(4)	1.250(19)
C(25)-C(26)	1.456(18)
C(26)-H(26A)	0.9800
C(26)-H(26B)	0.9800
C(26)-H(26C)	0.9800
C(23')-C(24')	1.44(2)
C(23')-H(23D)	0.9800
C(23')-H(23E)	0.9800
C(23')-H(23F)	0.9800
C(24')-O(3')	1.466(16)
C(24')-H(24C)	0.9900
C(24')-H(24D)	0.9900
O(3')-C(25')	1.293(18)
C(25')-O(4')	1.273(18)
C(25')-C(26')	1.506(18)
C(26')-H(26D)	0.9800
C(26')-H(26E)	0.9800
C(26')-H(26F)	0.9800

C(20)-O(1)-C(21)	113.94(15)
C(20)-O(2)-C(22)	113.97(15)
C(2)-C(1)-C(3)#1	122.83(15)
C(2)-C(1)-H(1)	118.6
C(3)#1-C(1)-H(1)	118.6
C(1)-C(2)-C(3)	118.63(13)
C(1)-C(2)-C(7)	119.05(14)
C(3)-C(2)-C(7)	122.32(13)
C(1)#1-C(3)-C(2)	118.55(14)
C(1)#1-C(3)-C(4)	118.55(14)
C(2)-C(3)-C(4)	122.90(13)
C(5)-C(4)-C(3)	112.11(13)
C(5)-C(4)-H(4A)	109.2
C(3)-C(4)-H(4A)	109.2
C(5)-C(4)-H(4B)	109.2
C(3)-C(4)-H(4B)	109.2
H(4A)-C(4)-H(4B)	107.9
C(6)-C(5)-C(4)	124.99(14)
C(6)-C(5)-C(19)	108.29(13)
C(4)-C(5)-C(19)	126.54(14)
C(5)-C(6)-C(7)	125.01(14)
C(5)-C(6)-C(8)	107.55(14)
C(7)-C(6)-C(8)	127.39(14)
C(6)-C(7)-C(2)	112.58(14)
C(6)-C(7)-H(7A)	109.1
C(2)-C(7)-H(7A)	109.1
C(6)-C(7)-H(7B)	109.1
C(2)-C(7)-H(7B)	109.1
H(7A)-C(7)-H(7B)	107.8
C(9)-C(8)-C(6)	105.59(12)
C(9)-C(8)-C(20)	98.39(13)
C(6)-C(8)-C(20)	98.54(13)
C(9)-C(8)-H(8)	117.1
C(6)-C(8)-H(8)	117.1
C(20)-C(8)-H(8)	117.1
C(10)-C(9)-C(18)	121.03(15)
C(10)-C(9)-C(8)	132.80(16)

C(18)-C(9)-C(8)	106.16(14)
C(9)-C(10)-C(11)	119.31(17)
C(9)-C(10)-H(10)	120.3
C(11)-C(10)-H(10)	120.3
C(12)-C(11)-C(16)	118.57(16)
C(12)-C(11)-C(10)	121.64(18)
C(16)-C(11)-C(10)	119.78(16)
C(13)-C(12)-C(11)	121.0(2)
C(13)-C(12)-H(12)	119.5
C(11)-C(12)-H(12)	119.5
C(12)-C(13)-C(14)	120.4(2)
C(12)-C(13)-H(13)	119.8
C(14)-C(13)-H(13)	119.8
C(15)-C(14)-C(13)	120.48(19)
C(15)-C(14)-H(14)	119.8
C(13)-C(14)-H(14)	119.8
C(14)-C(15)-C(16)	120.9(2)
C(14)-C(15)-H(15)	119.6
C(16)-C(15)-H(15)	119.6
C(15)-C(16)-C(11)	118.68(18)
C(15)-C(16)-C(17)	122.08(18)
C(11)-C(16)-C(17)	119.23(15)
C(18)-C(17)-C(16)	119.38(17)
C(18)-C(17)-H(17)	120.3
C(16)-C(17)-H(17)	120.3
C(17)-C(18)-C(9)	121.19(16)
C(17)-C(18)-C(19)	132.55(17)
C(9)-C(18)-C(19)	106.26(14)
C(18)-C(19)-C(5)	105.46(13)
C(18)-C(19)-C(20)	98.48(13)
C(5)-C(19)-C(20)	98.40(13)
C(18)-C(19)-H(19)	117.1
C(5)-C(19)-H(19)	117.1
C(20)-C(19)-H(19)	117.1
O(2)-C(20)-O(1)	110.92(13)
O(2)-C(20)-C(8)	117.55(14)
O(1)-C(20)-C(8)	108.41(14)
O(2)-C(20)-C(19)	109.28(13)

O(1)-C(20)-C(19)	116.16(13)
C(8)-C(20)-C(19)	93.81(13)
O(1)-C(21)-H(21A)	109.5
O(1)-C(21)-H(21B)	109.5
H(21A)-C(21)-H(21B)	109.5
O(1)-C(21)-H(21C)	109.5
H(21A)-C(21)-H(21C)	109.5
H(21B)-C(21)-H(21C)	109.5
O(2)-C(22)-H(22A)	109.5
O(2)-C(22)-H(22B)	109.5
H(22A)-C(22)-H(22B)	109.5
O(2)-C(22)-H(22C)	109.5
H(22A)-C(22)-H(22C)	109.5
H(22B)-C(22)-H(22C)	109.5
C(24)-C(23)-H(23A)	109.5
C(24)-C(23)-H(23B)	109.5
H(23A)-C(23)-H(23B)	109.5
C(24)-C(23)-H(23C)	109.5
H(23A)-C(23)-H(23C)	109.5
H(23B)-C(23)-H(23C)	109.5
C(23)-C(24)-O(3)	88.9(12)
C(23)-C(24)-H(24A)	113.8
O(3)-C(24)-H(24A)	113.8
C(23)-C(24)-H(24B)	113.8
O(3)-C(24)-H(24B)	113.8
H(24A)-C(24)-H(24B)	111.1
C(25)-O(3)-C(24)	121.1(15)
O(4)-C(25)-O(3)	113.8(15)
O(4)-C(25)-C(26)	120.5(17)
O(3)-C(25)-C(26)	123.0(17)
C(25)-C(26)-H(26A)	109.5
C(25)-C(26)-H(26B)	109.5
H(26A)-C(26)-H(26B)	109.5
C(25)-C(26)-H(26C)	109.5
H(26A)-C(26)-H(26C)	109.5
H(26B)-C(26)-H(26C)	109.5
C(24')-C(23')-H(23D)	109.5
C(24')-C(23')-H(23E)	109.5

H(23D)-C(23')-H(23E)	109.5
C(24')-C(23')-H(23F)	109.5
H(23D)-C(23')-H(23F)	109.5
H(23E)-C(23')-H(23F)	109.5
C(23')-C(24')-O(3')	102.0(13)
C(23')-C(24')-H(24C)	111.4
O(3')-C(24')-H(24C)	111.4
C(23')-C(24')-H(24D)	111.4
O(3')-C(24')-H(24D)	111.4
H(24C)-C(24')-H(24D)	109.2
C(25')-O(3')-C(24')	126.4(12)
O(4')-C(25')-O(3')	111.2(13)
O(4')-C(25')-C(26')	111.9(16)
O(3')-C(25')-C(26')	133.9(17)
C(25')-C(26')-H(26D)	109.5
C(25')-C(26')-H(26E)	109.5
H(26D)-C(26')-H(26E)	109.5
C(25')-C(26')-H(26F)	109.5
H(26D)-C(26')-H(26F)	109.5
H(26E)-C(26')-H(26F)	109.5

Symmetry transformations used to generate equivalent atoms:

#1 -x,-y+1,-z+1

Supplementary Table 4. Anisotropic displacement parameters (Å2x 103) for 5b.

The anisotropic displacement factor exponent takes the form: $-2\pi^2[h^2 a^{*2}U^{11} + ... + 2 h k a^* b^* U^{12}]$

	U^{11}	U^{22}	U^{33}	U^{23}	U^{13}	U^{12}
O(1)	26(1)	55(1)	47(1)	6(1)	8(1)	-11(1)
O(2)	33(1)	57(1)	34(1)	6(1)	1(1)	-2(1)
C(1)	25(1)	30(1)	33(1)	-4(1)	5(1)	-2(1)
C(2)	22(1)	33(1)	32(1)	-2(1)	5(1)	-2(1)
C(3)	24(1)	31(1)	30(1)	0(1)	4(1)	-4(1)
C(4)	26(1)	34(1)	40(1)	-4(1)	6(1)	-6(1)
C(5)	22(1)	38(1)	31(1)	-1(1)	2(1)	-5(1)
C(6)	21(1)	42(1)	31(1)	-3(1)	3(1)	-4(1)
C(7)	26(1)	41(1)	45(1)	-12(1)	10(1)	-5(1)
C(8)	22(1)	46(1)	31(1)	-3(1)	4(1)	-4(1)
C(9)	21(1)	46(1)	31(1)	-3(1)	4(1)	-7(1)
C(10)	23(1)	48(1)	35(1)	-2(1)	7(1)	-4(1)
C(11)	22(1)	59(1)	34(1)	3(1)	6(1)	-5(1)
C(12)	32(1)	68(1)	40(1)	7(1)	6(1)	4(1)
C(13)	40(1)	91(2)	40(1)	8(1)	0(1)	9(1)
C(14)	45(1)	106(2)	34(1)	-6(1)	-5(1)	4(1)
C(15)	40(1)	83(2)	36(1)	-12(1)	1(1)	-2(1)
C(16)	26(1)	62(1)	32(1)	-3(1)	4(1)	-6(1)
C(17)	29(1)	49(1)	37(1)	-7(1)	5(1)	-9(1)
C(18)	23(1)	42(1)	36(1)	-1(1)	5(1)	-9(1)
C(19)	24(1)	38(1)	38(1)	-3(1)	2(1)	-7(1)
C(20)	23(1)	50(1)	35(1)	2(1)	3(1)	-7(1)
C(21)	43(1)	60(1)	89(2)	5(1)	17(1)	-20(1)
C(22)	67(1)	86(2)	35(1)	2(1)	2(1)	15(1)
C(23)	97(8)	79(8)	110(8)	-21(6)	27(7)	-2(7)
C(24)	81(5)	66(6)	75(4)	-12(5)	42(4)	2(4)
O(3)	80(4)	57(5)	92(4)	-11(4)	44(3)	-4(4)
C(25)	74(4)	37(4)	86(5)	-13(4)	31(5)	-3(4)
O(4)	78(5)	83(5)	76(5)	-19(4)	-6(4)	3(4)
C(26)	91(6)	30(8)	57(6)	-9(5)	26(5)	-22(7)
C(23')	91(7)	17(7)	77(7)	2(5)	48(5)	-10(7)
C(24')	75(4)	47(4)	82(5)	-7(5)	41(5)	-2(4)
O(3')	77(4)	52(5)	81(3)	-6(4)	34(3)	-4(4)

C(25')	75(5)	62(6)	80(4)	-9(5)	48(4)	-9(5)
O(4')	85(5)	96(5)	64(4)	-16(4)	14(4)	-14(4)
C(26')	119(9)	90(7)	76(8)	21(6)	25(8)	-38(8)

Supplementary Table 5. Hydrogen coordinates (x 10^4) and isotropic displacement parameters ($Å^2$x 10 ^{3}) for **5b**.

	x	y	z	U(eq)
H(1)	314	2277	4347	35
H(4A)	945	9266	5069	40
H(4B)	1346	8310	5902	40
H(7A)	1916	3387	4742	45
H(7B)	1540	4288	3892	45
H(8)	3300	5458	4101	40
H(10)	4386	4041	5472	42
H(12)	5357	3192	6736	56
H(13)	6001	3952	8015	69
H(14)	5705	6829	8595	76
H(15)	4743	8917	7908	64
H(17)	3715	9772	6670	46
H(19)	2683	10450	5177	40
H(21A)	3813	11540	3730	95
H(21B)	4681	11327	4343	95
H(21C)	3810	11635	4673	95
H(22A)	3255	8876	2794	95
H(22B)	2257	8784	2480	95
H(22C)	2733	7010	2940	95
H(23A)	2477	4075	7598	141
H(23B)	2586	4496	6693	141
H(23C)	3274	3238	7255	141
H(24A)	2343	1355	6374	85
H(24B)	1587	2057	6844	85
H(26A)	2456	-2901	8527	87
H(26B)	2750	-962	8978	87
H(26C)	3337	-1947	8419	87
H(23D)	3349	-1191	8349	86
H(23E)	2547	-2401	8507	86
H(23F)	2633	-223	8771	86
H(24C)	1669	-705	7572	78
H(24D)	2464	-1354	7162	78

H(26D)	2392	4933	6275	141
H(26E)	2771	5219	7201	141
H(26F)	1769	5060	6920	141

Supplementary references

[i] Maeda, S.; Harabuchi, Y.; Sumiya, Y.; Takagi, M.; Suzuki, K.;Hatanaka, M.; Osada, Y.; Taketsugu, T.; Morokuma, K.; Ohno, K. GRRM17. http://iqce.jp/GRRM/index_e.shtml (accessed 2021-03-25).

[ii] Bruker, *SADABS*, Bruker AXS Inc., Madison, Wisconsin, USA, **2008**.

(1) Gaussian 16, Revision C.01, M. J. Frisch, G. W. Trucks, H. B. Schlegel, G. E. Scuseria, M. A. Robb, J. R. Cheeseman, G. Scalmani, V. Barone, G. A. Petersson, H. Nakatsuji, X. Li, M. Caricato, A. V. Marenich, J. Bloino, B. G. Janesko, R. Gomperts, B. Mennucci, H. P. Hratchian, J. V. Ortiz, A. F. Izmaylov, J. L. Sonnenberg, D. Williams- Young, F. Ding, F. Lipparini, F. Egidi, J. Goings, B. Peng, A. Petrone, T. Henderson, D. Ranasinghe, V. G. Zakrzewski, J. Gao, N. Rega, G. Zheng, W. Liang, M. Hada, M. Ehara, K. Toyota, R. Fukuda, J. Hasegawa, M. Ishida, T. Nakajima, Y. Honda, O. Kitao, H. Nakai, T. Vreven, K. Throssell, J. A. Montgomery, Jr., J. E. Peralta, F. Ogliaro, M. J. Bearpark, J. J. Heyd, E. N. Brothers, K. N. Kudin, V. N. Staroverov, T. A. Keith, R. Kobayashi, J. Normand, K. Raghavachari, A. P. Rendell, J. C. Burant, S. S. Iyengar, J. Tomasi, M. Cossi, J. M. Millam, M. Klene, C. Adamo, R. Cammi, J. W. Ochterski, R. L. Martin, K. Morokuma, O. Farkas, J. B. Foresman, and D. J. Fox, Gaussian, Inc., Wallingford CT, 2016.

(2) Pieniazek, S. N.; Clemente, F. R.; Houk, K. N. Sources of Error in DFT Computations of C[illegible]C Bond Formation Thermochemistries: Π→σ Transformations and Error Cancellation by DFT Methods. *Angew. Chem. Int. Ed.* **2008**, *47* (40), 7746–7749. https://doi.org/10.1002/anie.200801843.

(3) Jancarik, A.; Levet, G.; Gourdon, A. A Practical General Method for the Preparation of Long Acenes. *Chem. - Eur. J.* **2019**, *25* (9), 2366–2374. https://doi.org/10.1002/chem.201805975.

(4) Holec, J.; Cogliati, B.; Lawrence, J.; Berdonces-Layunta, A.; Herrero, P.; Nagata, Y.; Banasiewicz, M.; Kozankiewicz, B.; Corso, M.; Oteyza, D. G.; Jancarik, A.; Gourdon, A. A Large Starphene Comprising Pentacene Branches. *Angew. Chem. Int. Ed.* **2021**, anie.202016163. https://doi.org/10.1002/anie.202016163.

(5) Sheldrick, G. M. Crystal Structure Refinement with SHELXL. *Acta Crystallogr. Sect. C Struct. Chem.* **2015**, *71* (1), 3–8. https://doi.org/10.1107/S2053229614024218.
